# Innate immune sensing of dietary alcohol ignites inflammation to drive alcohol-related disease

**DOI:** 10.1126/sciadv.aea3979

**Published:** 2026-04-10

**Authors:** Yeonseo Jang, Hoeun Bae, SuHyeon Oh, Gyeongju Yu, Hyun Bae, Minh Quan Nguyen, Raghvendra Mall, Minjie Fu, Aritra Ghosh, Jihye Lee, Suhyun Kim, Seyun Shin, Nabukenya Mariam, Cheong Seok, Daesik Kim, SangJoon Lee, Si Ming Man, Rajendra Karki

**Affiliations:** ^1^ Department of Biological Sciences, College of Natural Sciences, Seoul National University, Seoul, 08826, Republic of Korea.; ^2^ Department of Biological Science, Ulsan National Institute of Science and Technology (UNIST), Ulsan, 44919, Republic of Korea.; ^3^ Center for Study of Emerging and Re-emerging Viruses, Korea Virus Research Institute, Institute for Basic Science (IBS), Daejeon, Republic of Korea.; ^4^ Biotechnology Research Center, Technology Innovation Institute, Abu Dhabi, 9639, United Arab Emirates.; ^5^ Division of Immunology and Infectious Diseases, The John Curtin School of Medical Research, The Australian National University, Canberra, 2601, Australia.; ^6^ Department of Precision Medicine, School of Medicine, Sungkyunkwan University, Suwon, 16419, Republic of Korea.; ^7^ Institute of Molecular Biology and Genetics (IMBG), Seoul National University, Seoul, Republic of Korea.

## Abstract

Alcohol consumption has short- and long-term impacts on physical and mental health. Although multiple host and environmental factors contribute to alcohol-related disease, the innate immune sensors that detect toxic signals from alcohol remain poorly defined. Here, we show that alcohol cooperates with sterile- or infection-induced interferon signaling to drive inflammatory cell death, cytokine release, and liver injury in humans and mice. We identified the pattern recognition receptor Z-DNA binding protein 1 (ZBP1) as a key innate immune sensor mediating pyroptosis, apoptosis, and necroptosis in response to combined ethanol and interferon stimulation. While interferon elevated ZBP1, ethanol suppressed adenosine deaminase acting on RNA 1 (ADAR1) expression. Together, interferon and ethanol activated JNK signaling to promote Z-RNA formation, triggering ZBP1. These findings reveal a mechanism by which alcohol and interferon converge to induce ZBP1-dependent inflammatory cell death and liver pathology, providing mechanistic insight and highlighting potential therapeutic targets for alcohol-related disease.

## Teaser

ZBP1 is activated by alcohol and interferon signaling, promoting inflammation and progression of alcoholic liver disease.

## INTRODUCTION

Alcohol is one of the most consumed drugs worldwide and a leading risk factor of morbidity and mortality. Chronic excessive consumption of alcohol leads to liver diseases, cancer, accelerated aging, cardiovascular disease, and mental health conditions (*1*–*6*). Inflammatory infiltrates and biomarkers and necrotic hepatocytes are commonly detected in patients with alcoholic liver diseases (*7*, *8*), with chronic inflammation promoting steatosis to cirrhosis in the liver (*9*). Infectious diseases further amplify the effects of alcohol to perpetuate the cycle of disease, with viral infections caused by hepatitis B virus, hepatitis C virus, or severe acute respiratory syndrome coronavirus 2 increasing the susceptibility to alcoholic liver diseases (*10*–*12*).

The innate immune system plays a major role in sensing and responding to pathogen-associated molecular patterns (PAMPs) and danger-associated molecular patterns (DAMPs), initiated by pattern recognition receptors that ultimately shape the outcomes of inflammatory diseases (*13*–*15*). Alcohol and its by-products are DAMPs that induce the production of reactive oxygen species, mitochondrial damage, hypoxia, oxidative stress, and endoplasmic reticulum stress, activating apoptosis and necroptosis (*16*–*22*). Toll-like receptor 4 (TLR4) and inflammasome signaling have been implicated in the development of alcohol-induced liver damage (*23*–*25*), but these signaling pathways alone do not fully explain the cell death and pathological hallmarks seen in alcoholic liver diseases. In addition, the governing immune sensors of alcohol triggering cell death have remained unknown.

In this study, we identified that ethanol coupled with underlying sterile- or infection-induced interferon (IFN) drives lytic inflammatory cell death and inflammation in humans and mice. The innate immune sensor triggering cell death is Z-DNA binding protein 1 (ZBP1), which is activated by c-Jun N-terminal kinase (JNK) pathways and Z-RNA production. ZBP1-mediated sensing of dietary alcohol and IFN triggered the development of alcoholic liver disease. Our findings provide immunological insights into the molecular mechanisms and therapeutic targets of alcohol-induced pathology and inflammation.

## RESULTS

### Coupling of alcohol and inflammation triggers innate immune cell death

**Fig. 1.**
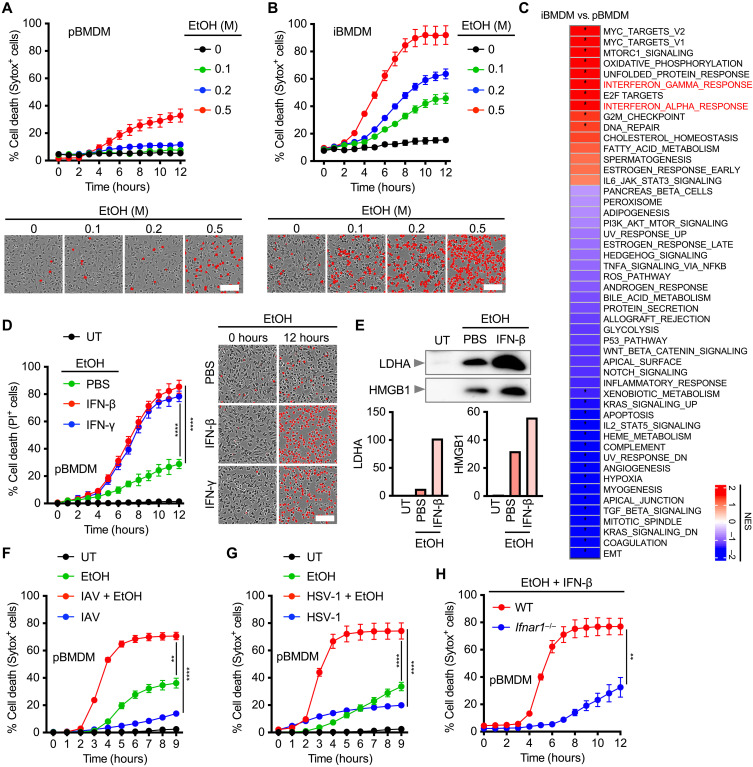
Alcohol and IFN induce cell death. (**A** and **B**) Real-time analysis of cell death in WT (A) pBMDMs and (B) iBMDMs treated with the indicated doses of ethanol (EtOH). The representative images of cell death (red color) at 9 hours are shown (below). Scale bars, 100 μm. (**C**) Heatmap depicting differentially regulated pathways in WT iBMDMs compared with pBMDMs. NES, normalized enrichment scores. (**D**) Real-time analysis of cell death in WT pBMDMs following stimulation with 0.6 M EtOH in the presence of PBS, IFN-γ (50 ng/ml), or IFN-β (20 ng/ml) added 12 hours before EtOH. The representative images of cell death (red color) at 12 hours are shown (right). Scale bar, 100 μm. (**E**) Immunoblot analysis of LDHA and HMGB1 in WT pBMDMs stimulated with EtOH plus IFN-β. Densitometric quantification of the representative blot is shown, relative to the untreated (UT) group. (**F** and **G**) Real-time analysis of cell death in WT pBMDMs infected with (F) IAV or (G) HSV-1 in the presence or absence of EtOH. (**H**) Real-time analysis of cell death in WT and *Ifnar1^−/−^* pBMDMs treated with EtOH plus IFN-β. Data are representative of at least two (C) or three [(A), (B), (D), (E), (F), (G), and (H)] independent experiments. *P < 0.05, **P < 0.01, and ****P < 0.0001. Analysis was performed using one-way analysis of variance (ANOVA) [(D), (F), and (G)] and *t* test (H). Data are shown as mean ± SEM [(A), (B), (D), (F), (G), and (H)].

To investigate the biological effects of alcohol, we treated primary bone marrow–derived macrophages (pBMDMs) and immortalized BMDMs (iBMDMs) with increasing concentrations of ethanol and monitored cell death. We noticed that iBMDMs were exquisitely sensitive to ethanol-induced cell death, whereas pBMDMs were largely resistant (Fig. 1, A and B). RNA sequencing (RNA-seq) analysis revealed highly enriched type I and type II IFN responses in iBMDMs relative to pBMDMs (Fig. 1C), suggesting increased IFN signaling as a possible amplifier of ethanol-induced cell death. Pretreating pBMDMs and hepatocytes, and human liver cell line HepG2, with either IFN-β or IFN-γ markedly elevated the sensitivity to alcohol-induced cell death across all cell types (Fig. 1D and fig. S1, A and B). We also observed a similar tendency in the release of inflammatory cytosolic contents, lactate dehydrogenase (LDH), and high mobility group box 1 (HMGB1) from pBMDMs, which occurs in response to a rupture of the plasma membrane (Fig. 1E). To examine the role of endogenous IFN, we infected cells with influenza A virus (IAV) and herpes simplex virus type 1 (HSV-1), both potent inducers of type I IFN responses (fig. S1, C and D). Consistent with exogenous IFN treatment, infection with IAV or HSV-1 rendered pBMDMs, HepG2 cells, and human monocytic leukemia cell line THP-1 highly sensitive to ethanol-induced cell death (Fig. 1, F and G, and fig. S1, E and F). These viruses also trigger the production of inflammatory cytokines through the TLR signaling (*26*, *27*). However, ethanol-induced cell death was not enhanced by other inflammatory ligands such as TNF-α or a TLR2 agonist, Pam3CSK4, but strongly potentiated by polyinosinic–polycytidylic acid [Poly(I:C)], a TLR3 agonist that triggers type I IFN signaling (fig. S1G). Following combined ethanol and IFN-β stimulation, pBMDMs and hepatocytes lacking the type I IFN receptor (*Ifnar1*^−/−^) were substantially more resistant to cell death and had diminished release of LDHA and HMGB1, as were HepG2 cells treated with an inhibitor of the JAK–signal transducers and activators of transcription pathways baricitinib (JAKi) (Fig. 1H and fig. S2, A to C). These data suggest that alcohol couples with sterile- or infection-induced IFN signaling to drive rapid lytic cell death in human and mouse cells.

**Fig. 2.**
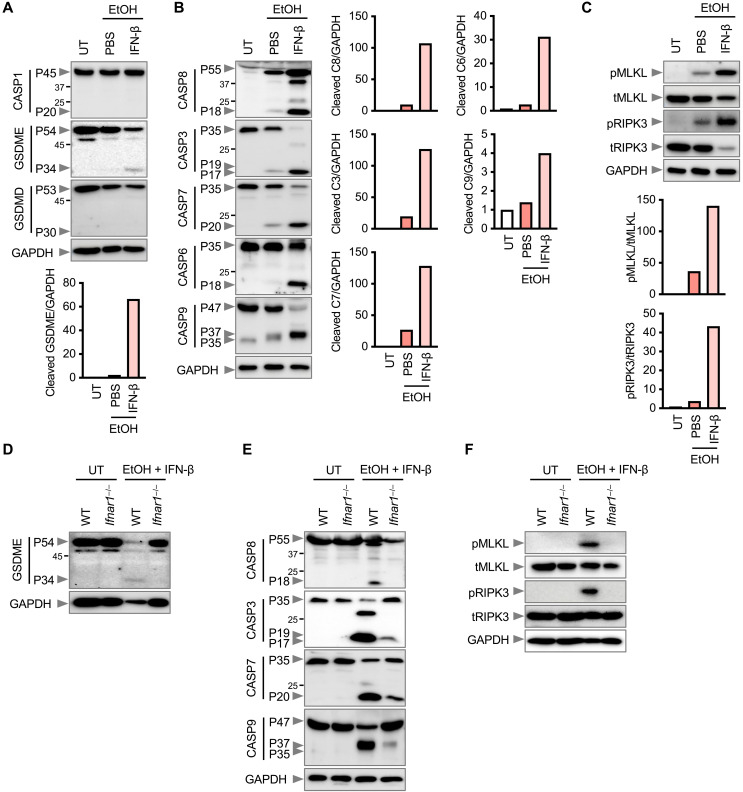
Alcohol and IFN promote activation of pyroptosis, apoptosis, and necroptosis. (**A** to **C**) Immunoblot analysis of (A) pro- (P45) and cleaved (P20) CASP1, pro- (P54) and activated (P34) GSDME, and pro- (P53) and activated (P30) GSDMD; (B) pro- (P55) and cleaved (P18) CASP8, pro- (P35) and cleaved (P19 and P17) CASP3, pro- (P35) and cleaved (P20) CASP7, pro- (P35) and cleaved (P18) CASP6, and pro- (P47) and cleaved (P37 and P35) CASP9; and (C) phosphorylated MLKL (pMLKL), total MLKL (tMLKL), phosphorylated RIPK3 (pRIPK3), and total RIPK3 (tRIPK3) in WT pBMDMs treated with 0.6 M EtOH in the presence of PBS or IFN-β (20 ng/ml). Densitometric quantification of the representative blot is shown, relative to the untreated (UT) group. (**D** to **F**) Immunoblot analysis of (D) GSDME; (E) CASP8, CASP3, CASP7, and CASP9; and (F) pMLKL, tMLKL, pRIPK3, and tRIPK3 in WT and *Ifnar1^−/−^* pBMDMs stimulated with EtOH plus IFN-β. Glyceraldehyde phosphate dehydrogenase (GAPDH) was used as an internal control. Data are representative of at least three independent experiments.

To investigate the mechanisms of cell death, we evaluated molecular markers of pyroptosis, apoptosis, and necroptosis. Wild-type (WT) pBMDMs treated with a combination of ethanol and IFN-β underwent proteolytic cleavage of the pyroptosis marker gasdermin E (GSDME), but not caspase-1 or GSDMD (Fig. 2A), and underwent the cleavage of the apoptosis markers caspase-3, caspase-6, caspase-7, caspase-8, and caspase-9 (Fig. 2B). Further, WT pBMDMs underwent phosphorylation of the necroptosis markers mixed lineage kinase domain-like protein (MLKL) and receptor-interacting protein kinase-3 (RIPK3) (Fig. 2C). Notably, ethanol alone in the absence of IFN-β did not robustly induce the activation of pyroptosis, apoptosis, and necroptosis markers (Fig. 2, A to C), reaffirming that induction of rapid cell death requires coupling of ethanol and IFN signaling (Fig. 1D). Combined ethanol and infection with IAV or HSV-1 also intensified the activation of pyroptosis, apoptosis, and necroptosis (fig. S2, D and E). We observed decreased activation of these inflammatory cell death molecules in *Ifnar1*^−/−^ pBMDMs stimulated with ethanol and IFN-β compared with WT pBMDMs (Fig. 2, D to F). However, genetic deletion of MLKL or GSDME, executioners of lytic cell death activated by alcohol and IFN, failed to protect from cell death (fig. S3, A and B). Pharmacological inhibition of caspases using the pan-caspase inhibitor Z-VAD-FMK (zVAD) to block pyroptosis and apoptosis, as well as inhibition of pyroptosis and necroptosis using the RIPK3 inhibitor GSK-872 in *Gsdme*^−/−^, did not inhibit cell death induced by ethanol and IFN-β (fig. S3, B and C). Inhibition of necroptosis alone using the RIPK1 inhibitor necrostatin-1 (Nec-1) partially blocked cell death induced by ethanol and IFN-β (fig. S3C) but completely blocked necroptosis induced by TNF-α, Smac mimetic LCL161, and zVAD (fig. S3D). A combination of zVAD and Nec-1 to inhibit pyroptosis, apoptosis, and necroptosis attenuated cell death (fig. S3C). Moreover, ethanol has been implicated in ferroptosis (*22*, *28*) and was found to induce lipid peroxidation, a hallmark of ferroptotic activity (fig. S3E). However, IFN treatment did not potentiate ethanol-induced lipid peroxidation (fig. S3E), and treatment with ferrostatin-1 (Fer-1), an inhibitor of lipid peroxidation, failed to prevent cell death induced by ethanol and IFN (fig. S3F). These data collectively suggest that ethanol, in conjunction with sterile- or infection-related IFN, drives mixed lineage lytic cell death involving pyroptosis, apoptosis, necroptosis, and ferroptosis, across human and mouse immune and nonimmune cells.

### Coupling of alcohol and IFN exacerbates alcoholic liver disease

**Fig. 3.**
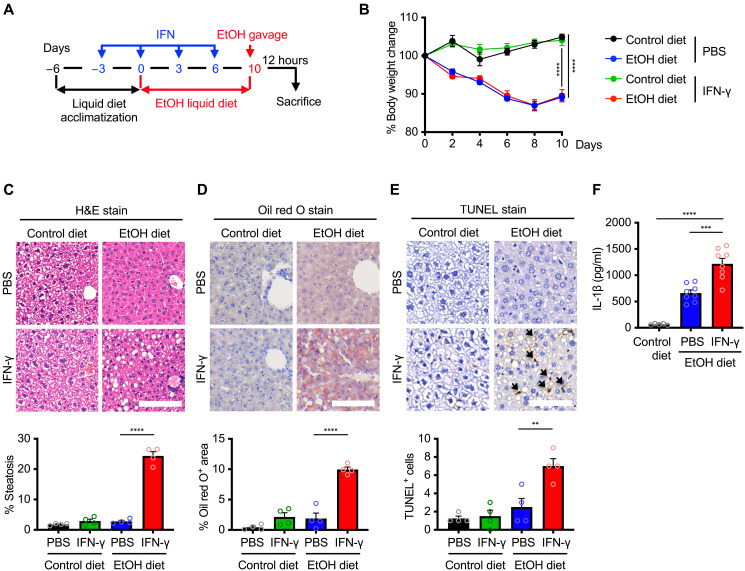
Alcohol and IFN drive the development of alcoholic liver disease. (**A**) Experimental design of the acute-on-chronic alcoholic liver injury model in mice, using EtOH (5% v/v) liquid diet and IFN injection (1 μg per mouse), followed by EtOH (5 g/kg) by oral gavage. (**B**) Body weight change of WT mice administered with control diet and vehicle PBS injection (*n* = 9), EtOH diet and vehicle PBS injection (*n* = 9), control diet and IFN-γ injection (*n* = 9), and EtOH diet and IFN-γ injection (*n* = 9). (**C** to **E**) Histopathological analysis, (C) hematoxylin and eosin (H&E) stain; (D) Oil red O stain; and (E) TUNEL stain of liver tissues from control- or EtOH-fed WT mice injected with PBS or IFN-γ. Black arrows indicate TUNEL^+^ cells. Scale bars, 400 μm (C) and 600 μm [(D) and (E)]. Quantifications of histopathological analyses are shown (below). (**F**) Analysis of the serum level of IL-1β in the blood of WT mice with control diet (*n* = 5), EtOH diet with PBS injection (*n* = 8), and EtOH diet with IFN-γ injection (*n* = 8). Data are pooled from two independent experiments [(B) to (F)]. **P < 0.01, ***P < 0.001, and ****P < 0.0001. Analysis was performed using two-way ANOVA (B) and one-way ANOVA [(C) to (F)]. Data are shown as mean ± SEM [(B) to (F)].

To uncover the physiological relevance of dietary alcohol and IFN in initiating or perpetuating disease, we adapted an established murine model of alcoholic liver disease. Mice were injected with recombinant IFN-γ on days −3, 0, 3, and 6 during 10 days of ethanol feeding (Fig. 3A). On day 10, mice were given a single binge ethanol feeding to induce liver injury, inflammation, and fatty liver, mimicking acute-on-chronic alcoholic liver injury in humans (Fig. 3A) (*29*). Mice receiving ethanol exhibited 10% loss of body weight over the 10-day feeding period, regardless of IFN-γ treatment (Fig. 3B). Histopathological assessments revealed progressive liver injury in mice given combined ethanol and IFN-γ, characterized by liver cell ballooning, cord derangement, Mallory-Denk bodies, intracellular space dilation, and inflammatory cell infiltration (Fig. 3, C to E). In contrast, the livers from mice left untreated, treated only with ethanol, or treated only with IFN-γ had reduced histological hallmarks (Fig. 3, C to E). In mice treated with combined ethanol and IFN-γ, Oil red O staining and terminal deoxynucleotidyl transferase–mediated deoxyuridine triphosphate nick end labeling (TUNEL) staining revealed liver steatosis and an increased number of dead cells, respectively (Fig. 3, D and E). In addition, these mice had increased proinflammatory cytokine interleukin-1β (IL-1β) production than mice treated only with ethanol (Fig. 3F).

**Fig. 4.**
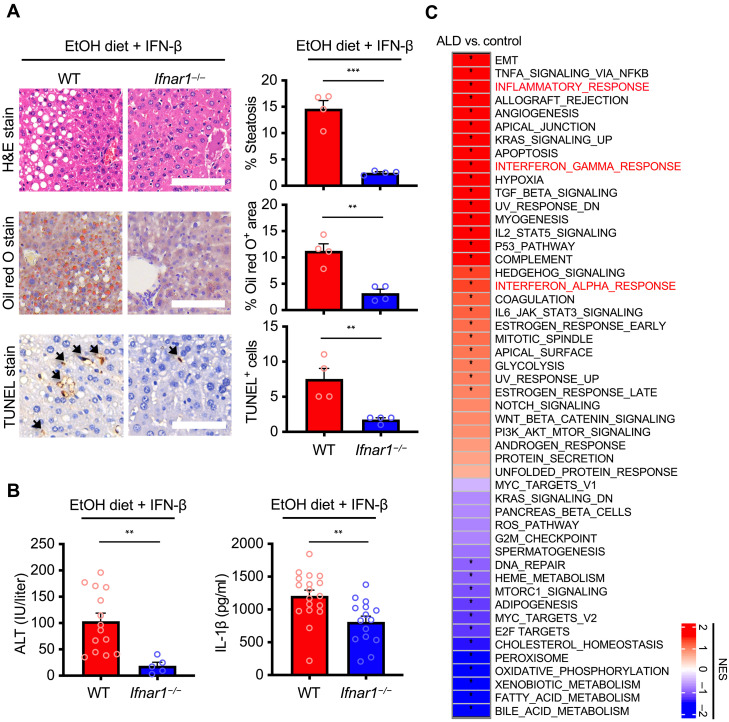
Loss of IFN signaling alleviates liver pathology in mice and patients with alcoholic liver disease have up-regulated signature of IFN. (**A**) Histopathological analysis, H&E, Oil red O, and TUNEL stain of liver tissues from WT and *Ifnar1^−/−^* mice fed with EtOH diet and injected with IFN-β. Black arrows indicate TUNEL^+^ cells. Scale bars, 400 μm (H&E) and 600 μm (Oil red O and TUNEL). Quantifications of histopathological analyses are shown (right). (**B**) Analysis of serum ALT in WT (*n* = 14) and *Ifnar1^−/−^* (*n* = 5) mice and IL-1β in WT (*n* = 18) and *Ifnar1^−/−^* (*n* = 15) mice fed with EtOH diet and injected with IFN-β. (**C**) Heatmap depicting differentially regulated pathways in liver transcriptomes of patients with alcoholic liver disease (ALD) compared with healthy people (control). Data are pooled from two independent experiments. *P < 0.05, **P < 0.01, and ***P < 0.001. Analysis was performed using *t* test and data are shown as mean ± SEM [(A) and (B)].

To provide further physiological insights, we treated WT and *Ifnar1*^−/−^ mice with ethanol and IFN-β to elucidate the role of interferon-alpha/beta receptor (IFNAR) signaling in alcoholic liver disease. Histopathological analysis revealed that *Ifnar1*^−/−^ mice had less microvesicular and macrovesicular steatosis and inflammatory cell recruitment in the livers compared with WT mice (Fig. 4A). Further, the amount of TUNEL^+^ cells and levels of the biomarker of liver damage, serum alanine transaminase (ALT), and IL-1β were substantially lower in *Ifnar1*^−/−^ mice than those in WT mice (Fig. 4, A and B). Using pathway analysis to identify transcriptomic changes between healthy people and those with alcoholic liver disease (*30*), we saw a notable enrichment of type I and type II IFN responses and inflammatory pathways from the livers of patients with alcoholic liver disease (Fig. 4C). We also found that patients with alcoholic liver disease had substantial levels of pyroptosis, apoptosis, and necroptosis biomarkers but not healthy individuals or those with nonalcoholic fatty liver disease, also known as NAFLD (fig. S4, A and B).

We next evaluated the functional contribution of necroptosis in alcoholic liver injury by administering ethanol and IFN-γ to WT and *Mlkl*^−/−^ mice (Fig. 3A). Histopathological and biochemical analyses revealed comparable degrees of hepatic steatosis, hepatocyte ballooning, Mallory-Denk body formation, and serum ALT levels between the two genotypes (fig. S4, C and D). These data indicate that necroptosis alone does not account for alcohol-induced liver injury, suggesting the involvement of additional inflammatory cell death pathways in the pathogenesis of alcoholic liver disease. Together, our data from both murine models and analyses of human patient demonstrate that alcohol consumption, in concert with IFN signaling, sensitizes cells to inflammatory cell death, thereby exacerbating the progression of alcoholic liver disease.

### ZBP1 is a sensor of alcohol and IFN

**Fig. 5.**
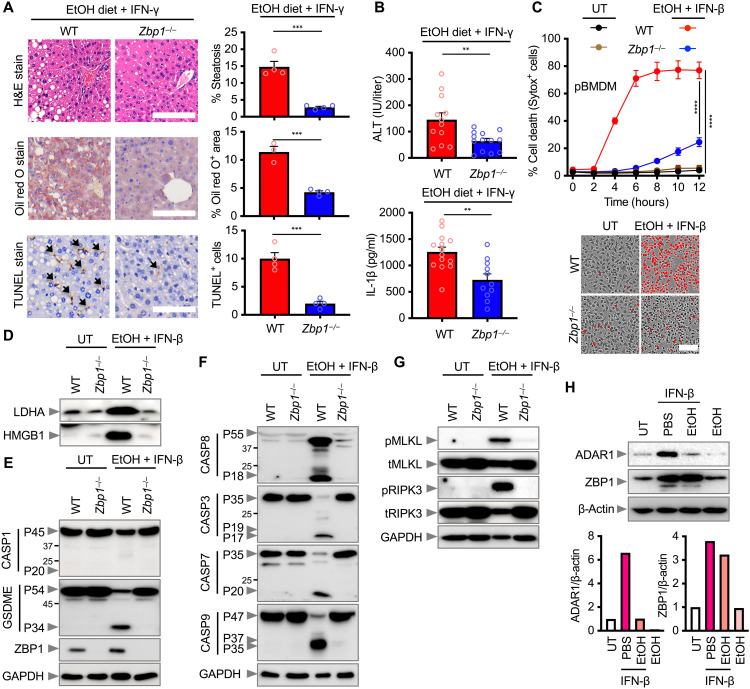
ZBP1 senses alcohol and IFN, driving alcoholic liver disease. (**A**) Histopathological analysis, H&E, Oil red O, and TUNEL stain of liver tissues from WT and *Zbp1^−/−^* mice fed with EtOH diet and injected with IFN-γ. Black arrows indicate TUNEL^+^ cells. Scale bars, 400 μm (H&E) and 600 μm (Oil red O and TUNEL). Quantifications of histopathological analyses are shown (right). (**B**) Analysis of serum ALT in WT (*n* = 12) and *Zbp1^−/−^* (*n* = 13) mice and IL-1β in WT (*n* = 14) and *Zbp1^−/−^* (*n* = 11) mice upon EtOH and IFN-γ administration. (**C**) Real-time analysis of cell death in WT and *Zbp1^−/−^* pBMDMs treated with 0.6 M EtOH plus IFN-β (20 ng/ml). Representative images of cell death (red color) at 9 hours are shown (below). Scale bar, 100 μm. (**D** to **G**) Immunoblot analysis of (D) LDHA and HMGB1; (E) pro-(P45) and cleaved (P20) CASP1, pro- (P54) and activated (P34) GSDME and ZBP1; (F) pro- (P55) and cleaved (P18) CASP8, pro- (P35) and cleaved (P19 and P17) CASP3, pro- (P35) and cleaved (P20) CASP7, and pro- (P47) and cleaved (P37 and P35) CASP9; and (G) pMLKL, tMLKL, pRIPK3, and tRIPK3 in WT and *Zbp1^−/−^* pBMDMs stimulated with EtOH plus IFN-β. (**H**) Immunoblot analysis of ADAR1 and ZBP1 in WT pBMDMs treated with EtOH, IFN-β, or a combination of both. Densitometric quantification of the representative blot is shown, relative to the untreated (UT) group. GAPDH or β-actin was used as an internal control. Data are representative of at least two independent experiments. **P < 0.01, ***P < 0.001, and ****P < 0.0001. Analysis was performed using *t* test [(A) and (B)] and two-way ANOVA (C). Data are shown as mean ± SEM [(A) to (C)].

Innate immunity is the first line of defense against PAMPs and DAMPs, potentially leading to lytic cell death (*31*, *32*). We sought to identify the specific innate immune sensor driving inflammatory cell death triggered by ethanol and IFN. Among the IFN-stimulated genes, we found the up-regulation of the gene encoding the innate immune sensor ZBP1 in patients with alcoholic liver disease (fig. S5A) but not in healthy individuals or individuals with NAFLD (fig. S5B). To this end, we administered WT and *Zbp1*^−/−^ mice with ethanol and IFN-γ and observed remarkable resistance of *Zbp1*^−/−^ mice to progressive liver injury, along with less microsteatosis, hepatocyte ballooning, and Mallory-Denk bodies in the liver compared with WT mice (Fig. 5A). The presence of TUNEL^+^ dead cells, infiltration of inflammatory cells, and levels of ALT and IL-1β were all reduced in *Zbp1*^−/−^ mice compared with those in WT mice (Fig. 5, A and B). In addition, activation of inflammatory cell death pathways triggered by ethanol was suppressed in the liver tissue of *Zbp1*^−/−^ mice compared to WT mice (fig. S5C). Further, we treated WT and *Zbp1*^−/−^ pBMDMs with ethanol and IFN-β and found that *Zbp1*^−/−^ pBMDMs were markedly more resistant to cell death, whereas WT pBMDMs underwent rapid cell death (Fig. 5C). *Zbp1*^−/−^ pBMDMs were also less susceptible to releasing LDHA and HMGB1 compared with WT pBMDMs (Fig. 5D). Compared with WT pBMDMs, *Zbp1*^−/−^ pBMDMs had an impaired ability to undergo proteolytic cleavage of the pyroptosis marker GSDME and the apoptosis markers caspase-3, caspase-7, caspase-8, and caspase-9 (Fig. 5, E and F). *Zbp1*^−/−^ pBMDMs also had an impaired ability to undergo phosphorylation of the necroptosis markers MLKL and RIPK3 (Fig. 5G). In contrast, a loss of ZBP1 failed to prevent lipid peroxidation induced by ethanol plus IFN-β (fig. S5D). Consistent with this, both WT and *Zbp1*^−/−^ pBMDMs exhibited comparable levels of cell death following treatment with the ferroptosis activator RSL3 (fig. S5E). These observations suggest that ZBP1 is essential for the execution of pyroptosis, apoptosis, and necroptosis triggered by ethanol plus IFN-β but is not required for lipid peroxidation and ferroptosis. Notably, ZBP1 expression increased following IFN-β stimulation and remained elevated upon ethanol treatment (Fig. 5H), which aligns with synergistic induction of cell death by ethanol and IFN. Although ZBP1 is associated with absent in melanoma 2 (AIM2) and stimulator of interferon genes (STING)-mediated responses (*33*, *34*), these nucleic acid sensors were not required for the induction of cell death in response to ethanol and IFN-β (fig. S6, A and B). These data collectively suggest that ZBP1 is the innate immune sensor of ethanol and IFN that ignites inflammatory cell death in alcoholic liver disease.

### Alcohol and IFN generate Z-RNA via JNK to activate ZBP1

**Fig. 6.**
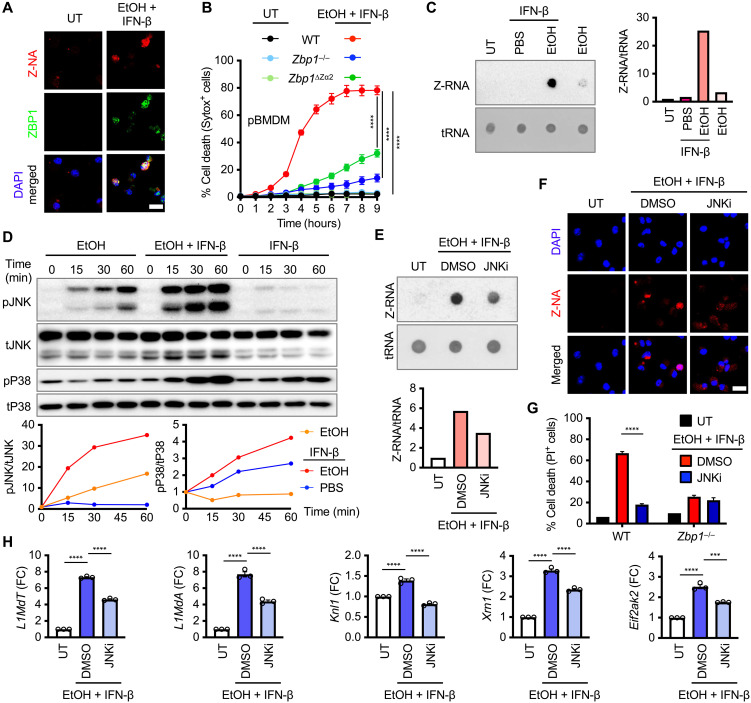
Alcohol and IFN activate JNK signaling and the formation of Z-RNA. (**A**) Immunofluorescence images of WT pBMDMs treated with 0.6 M EtOH plus IFN-β (20 ng/ml) for 3 hours. Scale bar, 15 μm. (**B**) Real-time analysis of cell death in WT, *Zbp1^−/−^*, and *Zbp1*^∆*Z*α*2*^ pBMDMs treated with EtOH plus IFN-β. (**C**) Immuno-dot blot analysis of Z-RNA in WT pBMDMs treated with EtOH, IFN-β, or a combination of both for 3 hours. (**D**) Immunoblot analysis of phosphorylated JNK (pJNK), total JNK (tJNK), phosphorylated p38 (pP38), and total p38 (tP38) in WT pBMDMs treated with EtOH, IFN-β, or a combination of both for the indicated time. (**E**) Immuno-dot blot analysis of Z-RNA in WT pBMDMs treated with 1 μM JNK-IN-8 (JNKi) upon treatment of EtOH plus IFN-β. (**F**) Immunofluorescence images of pBMDMs treated with EtOH plus IFN-β for 3 hours, in the presence or absence of JNKi. Scale bar, 15 μm. DMSO, dimethyl sulfoxide. (**G**) Percentage of cell death in WT and *Zbp1^−/−^* pBMDMs treated with JNKi upon EtOH plus IFN-β treatment. (**H**) Representative data of quantitative real-time PCR analysis of genes (*L1MdT*, *L1MdA*, *Knl1*, *Xrn1*, and *Eif2ak2*) in WT pBMDMs treated with JNKi upon EtOH plus IFN-β stimulation. *Gapdh* was used as an internal control. Data are representative of at least three independent experiments. Densitometric quantification of the representative blot is shown, relative to untreated (UT) groups [(C) and (E)] or the 0-min group (D). ***P < 0.001 and ****P < 0.0001. Analysis was performed using two-way ANOVA [(B) and (G)] and one-way ANOVA (H). Data are shown as mean ± SEM [(B), (G), and (H)].

Alcohol and IFN may have liberated cytoplasmic ligands that directly activate ZBP1. We and others have previously shown that ZBP1 can sense Z-nucleic acid (Z-NA) through the Zα2 domain (*35*, *36*), and oxidative stress can trigger the accumulation of Z-NA (*37*, *38*). We hypothesized that alcohol-induced oxidative stress promotes the formation and accumulation of Z-NA. Cotreatment of ethanol and IFN-β strongly increased cytosolic Z-NA and ZBP1 signals, which exhibited strong colocalization (Fig. 6A). Primary BMDMs lacking the ZBP1 Zα2 domain (*Zbp1*^∆Zα2^ pBMDMs) were largely resistant to cell death triggered by ethanol and IFN-β compared with WT pBMDMs (Fig. 6B). Notably, ribonuclease A (RNase A) treatment abolished the Z-NA accumulation induced by ethanol and IFN-β (fig. S7, A and B), indicating that Z-NA arises from Z-RNA rather than Z-DNA. These data suggest that alcohol and IFN generate Z-RNA and activate ZBP1-dependent cell death.

While cotreatment of ethanol and IFN-β induced robust Z-RNA formation, ethanol or IFN-β alone showed minimal or no detectable signal (Fig. 6C). As IFN-β has been reported to induce Z-RNA accumulation when adenosine deaminase acting on RNA 1 (ADAR1) is depleted (*39*), we examined whether alcohol alters IFN-β–mediated ADAR1 induction. Ethanol markedly reduced ADAR1 protein levels without affecting *Adar1* mRNA and had no effect on ZBP1 expression (Fig. 5H and fig. S7C). Together, these findings indicate that alcohol promotes posttranscriptional loss of ADAR1, thereby facilitating IFN-induced Z-RNA accumulation.

Oxidative stress engages multiple stress-activated signaling cascades, including JNK and p38 mitogen-activated protein kinase (MAPK) pathways, which are associated with cell death (*40*, *41*). Given that alcohol induces oxidative stress (fig. S3E), we checked whether these kinases contribute to Z-RNA accumulation and ZBP1-dependent inflammatory cell death. Treatment with ethanol alone or IFN-β alone exhibited minimal or modest phosphorylation of JNK and p38, while cotreatment of ethanol and IFN-β triggered robust activation of both kinases (Fig. 6D). Pharmacological inhibition of JNK using JNK-IN-8 (*42*) markedly reduced cytoplasmic Z-RNA accumulation (Fig. 6, E and F) and attenuated cell death (Fig. 6G). Moreover, the addition of JNK-IN-8 to *Zbp1*^−/−^ pBMDMs did not further decrease cell death (Fig. 6G), implying that JNK and ZBP1 operate within the same pathway to induce cell death.

To identify the endogenous sources of Z-RNA generated from JNK signaling, we treated pBMDMs with ethanol and IFN-β in the presence or absence of JNK-IN-8. We analyzed representative ADAR1-targeted genes, including retroelements and IFN-stimulated genes (*39*). Transcripts of the murine long interspersed nuclear element-1 (LINE-1) retrotransposons *L1MdT* and *L1MdA*, as well as *Knl1*, *Xrn1*, *Eif2ak2*, *Slfn5*, *Ifih1*, *Ddx58*, and *Isg15*, were induced by combined alcohol and IFN-β treatment (Fig. 6H and fig. S7D). JNK inhibition selectively attenuated the up-regulation of *L1MdT* and *L1MdA*, and to some extent, *Knl1*, *Xrn1*, and *Eif2ak2* (Fig. 6H), while other genes remained unaffected (fig. S7D). To determine whether LINE-1–encoded transcripts adopt the Z-NA conformation, we performed RNA immunoprecipitation (RIP) using the Z22 antibody followed by quantitative polymerase chain reaction (PCR). LINE-1–encoded transcripts were enriched in Z22 immunoprecipitates compared with immunoglobulin G (IgG) controls from pBMDMs treated with alcohol and IFN-β, with the enrichment diminished by JNK inhibition (fig. S7E). These findings suggest that alcohol and IFN activate the JNK pathway, which enhances the transcription of Z-RNA–forming elements. In conclusion, ethanol and IFN cooperatively induce endogenous Z-RNA through JNK signaling, and ZBP1 senses this Z-RNA via its Zα2 domain to trigger inflammatory cell death (fig. S8).

## DISCUSSION

Our study identifies ZBP1 as an innate immune sensor that integrates signals from alcohol and IFN to drive inflammatory lytic cell death, hepatic inflammation, and liver pathology. As a central mediator of cell death, ZBP1 coordinates multiple, interconnected cell death pathways (*35*, *43*, *44*). In line with this, ethanol plus IFN-induced cell death was not abolished by the deletion of a single executioner. However, protection achieved through combined inhibition of multiple cell death effectors suggests that ethanol and IFN engage several converging mechanisms rather than a single dominant pathway. These findings highlight the pivotal role of ZBP1 as an upstream integrator of multiple lytic cell death pathways. Given that ZBP1 is transcriptionally induced by IFNs, the role of IFN as a priming inflammatory signal that ignites alcohol-induced cell death is of particular interest. IFN signaling can arise from sterile inflammatory conditions, such as autoimmunity, or during microbial and viral infections. Accordingly, alcohol exposure in the context of infection or preexisting inflammatory conditions represents a high-risk setting for the development and progression of alcoholic liver disease (*10*–*12*). In such contexts, lytic cell death is driven by IFN-dependent signaling.

At the molecular level, ethanol disrupted the homeostatic balance between ADAR1 and ZBP1, two key regulators of Z-NA sensing (*45*). We found that alcohol suppressed ADAR1 induction during IFN signaling without affecting ZBP1 expression. Since ADAR1 limits ZBP1 activation by editing endogenous Z-RNA or by preventing ZBP1-RIPK3 complex formation (*35*, *39*), the alcohol-mediated down-regulation of ADAR1 likely removes this regulatory brake, thereby amplifying ZBP1-dependent inflammatory cell death in the presence of IFN. Mechanistically, alcohol and IFN converge on the JNK signaling pathway to promote Z-RNA formation. Among the transcripts examined, the LINE-1–encoded retroelements *L1MdT* and *L1MdA* were prominently induced under ethanol and IFN stimulation, and their expression was markedly reduced by JNK inhibition. Other ADAR1-regulated transcripts—such as *Knl1*, *Xrn1*, and *Eif2ak2*—were modestly affected, suggesting that LINE-1 elements are major contributors to Z-RNA accumulation, although additional sources are likely involved. Given the complexity of stress-responsive signaling and RNA metabolism, other pathways beyond JNK may also contribute to Z-RNA generation and subsequent ZBP1 activation during alcohol and IFN exposure.

In conclusion, our findings reveal a previously unrecognized alcohol-IFN-ZBP1 axis that links metabolic stress and inflammation to innate immune sensing and inflammatory cell death. This mechanism provides insights into how chronic alcohol consumption and inflammatory signaling synergize to promote liver injury and may offer potential avenues of prevention or treatment of alcoholic liver diseases by targeting ZBP1-mediated pathways.

## MATERIALS AND METHODS

### Mice

C57BL/6J (WT) mice were purchased from Raonbio (Yongjin, Korea). *Ifnar1*^−/−^ and *Zbp1*^−/−^ mice on a C57BL/6J background were purchased from the Jackson Laboratory (stock number 028288) and Cyagen (*46*), respectively. *Aim2*^−/−^ mice were provided by S. Lee [Ulsan National Institute of Science and Technology (UNIST)]. *Gsdme*^−/−^ mice were provided by S. M. Man (Australian National University). *Mlkl*^−/−^ mice were provided by J. Song (Yonsei University). *Sting*^−/−^ mice were provided by J. Park [Seoul National University (SNU)]. *Zbp1*^Δ*Z*α*2*^ mice were received from T.-D. Kanneganti (St. Jude Children’s Research Hospital) (*47*).

Mice were group-housed (up to five mice per cage), bred under standard pathogen-free housing conditions in a 12-hour light/dark cycle (lights on from 6 a.m. to 6 p.m.), and fed standard chow in the animal facility at SNU and UNIST. Both male and female mice were used. Age- and sex-matched 6- to 8-week-old mice were used for in vitro work, and 10- to 12-week-old mice were used for in vivo work. Cohoused animals were used for in vivo analysis. Experimental procedures for in vivo were conducted in accordance with protocols approved by the Institutional Animal Care and Use Committee of SNU (SNU-241108-1-1) and UNIST (UNISTIACUC-24-080).

### Generation of iBMDMs

WT primary BMDMs were transformed according to the established protocol (*48*). Briefly, BMDMs were exposed to the J2Cre virus for 48 hours, followed by a gradual reduction in L-cell–conditioned medium over a period of 6 to 10 weeks.

### Isolation of primary hepatocytes

Primary hepatocytes were isolated from mice as described previously (*49*). Mouse livers were subjected to perfusion, and liver cells were dissociated using collagenase. Subsequently, hepatocytes were isolated using density gradient centrifugation.

### Cell culture

Primary BMDMs were derived from the bone marrow of WT and mutant mice. Cells were cultivated for 6 days in Dulbecco’s modified Eagle’s medium (DMEM; Biowest, L0103-500) supplemented with 30% L929-conditioned medium, 10% fetal bovine serum (FBS; Biowest, S1480), 1% penicillin and streptomycin (Biowest, L0022-100), and 1% non-essential amino acids (Biowest, X0577-100) (*50*). Primary BMDMs were seeded in 12-well plates (1× 10^6^ cells per well) or 24-well plates (5 × 10^5^ cells per well) and incubated overnight before stimulation. iBMDMs were grown in DMEM supplemented with 3% L929-conditioned medium, 10% FBS, 1% penicillin and streptomycin, and 1% non-essential amino acids and seeded in 24-well plates (2.5 × 10^5^ cells per well). THP-1 cells (gift from A. Kawaguchi, University of Tsukuba) were grown in RPMI 1640 (Biowest, L0498) with 5% FBS and differentiated into macrophages in RPMI 1640 medium containing 5% FBS and phorbol 12-myristate 13-acetate (100 ng/ml) for 2 days and seeded in 24-well plates (5 × 10^5^ cells per well). HepG2 cells (Korean Cell line Bank, 88065) were grown in DMEM (Thermo Fisher Scientific, 11995065) supplemented with 5% FBS and 1% penicillin-streptomycin and seeded in 24-well plates (2.5 × 10^5^ cells per well). Primary hepatocytes suspended in DMEM with 10% FBS and 1% penicillin-streptomycin were seeded in collagen-coated 12-well plates (2 × 10^5^ cells per well).

### Virus culture

Human HSV-1 (HF strain) (American Type Culture Collection, VR-260) was purchased and propagated in Vero cells (Korean Cell Line Bank, 10081). The virus titer was quantified using the plaque assay in Vero cells. IAV (A/Puerto Rico/8/34, H1N1, also known as PR8) was provided by M.-S. Park (Korea University) and propagated in 11-day-old embryonated chicken eggs via allantoic inoculation. The IAV titer was measured using the plaque assay in Madin-Darby canine kidney cells (gift from A. Kawaguchi, University of Tsukuba).

### Cell stimulation and infection

Cells were stimulated with the following specified concentrations of cell death triggers, unless stated otherwise: 0.6 M ethanol (Sigma-Aldrich, 459844); cotreatment with TNF-α (75 ng/ml; PeproTech, 315-01A), 200 nM Smac mimetic LCL161 (Selleckchem, S7009), and 25 μM Z-VAD-FMK (Selleckchem, S7023) which triggers necroptosis; 1 μM RSL3 (Selleckchem, S8155) which triggers ferroptosis. To prevent ethanol evaporation, plates were sealed with a film (Axygen, PCR-SP). For priming, cells were added with IFN-β (20 ng/ml; PBL assay science, 12400-1), IFN-γ (50 ng/ml; PeproTech, 315-05), TNF-α (50 ng/ml), Pam3CSK4 (1 μg/ml; InvivoGen, tlrl-pms), or Poly(I:C) (5 μg/ml; InvivoGen, tlrl-pic) for 12 hours before treatment with cell death triggers. For inhibitor treatment, 1 μM JNK-IN-8 (Selleckchem, S4901) was added to cells 12 hours prior, and other inhibitors were added 1 hour before treating cell death triggers: 5 μM baricitinib (MedChemExpress, HY-15315), 25 μM Z-VAD-FMK and 5 μM GSK-872 (Selleckchem, S8465), 40 μM necrostain-1 (Selleckchem, S8037), and 10 μM ferrostatin-1 (Sigma-Aldrich, SML0583). For infections, cells were infected with viruses in high-glucose DMEM (WELGENE, LM001-03) 6 hours before ethanol treatment: 5 multiplicity of infection (MOI; pBMDMs and THP-1) and 20 MOI (HepG2) of HSV-1 or 2 MOI (pBMDMs), 4 MOI (HepG2), and 10 MOI (THP-1) of IAV.

### Real-time analysis of cell death and lipid peroxidation

Real-time cell death assays were performed on an IncuCyte system (Sartorius, IncuCyte SX5). Cells were seeded in 12-well (primary hepatocytes) or 24-well (pBMDMs, iBMDMs, THP-1, and HepG2) plates and stimulated. SYTOX Green (Thermo Fisher Scientific, S7020) or propidium iodide (Invitrogen, P3566) was added at the time of stimulation for cell death analysis. Images (four image fields per well) were acquired every 1 hour at 37°C and 5% CO_2_. Subsequent image analysis was conducted using the software package supplied with the IncuCyte imager; the number of SYTOX Green–positive cells (SYTOX^+^ cells) or propidium iodide–positive cells (PI^+^ cells) present in each image was counted. For lipid peroxidation analysis, BODIPY 581/591 C11 (Thermo Fisher Scientific, D3861) was added at the time of stimulation according to the manufacturer’s instructions, and representative images were acquired at the indicated time points.

### Immunoblot analysis

Immunoblotting was performed as described previously (*51*). For caspase analysis, cells were lysed along with the supernatant using 50 μl of caspase lysis buffer (containing 1× protease inhibitors, 1× phosphatase inhibitors, 10% NP-40, and 25 mM dithiothreitol), followed by the addition of 100 μl of 4× sample loading buffer (containing SDS and 2-mercaptoethanol). To analyze LDHA and HMGB1, cell culture supernatant samples were centrifuged (8000 rpm for 4 min), and 180 μl of the supernatant was combined with 60 μl of sample loading buffer. To analyze signaling proteins, cell culture supernatants were removed at the indicated time points, and the cells were washed once with phosphate-buffered saline (PBS), followed by lysis with 150 μl of radioimmunoprecipitation assay buffer (containing 1× phosphatase inhibitor, 1/2× protease inhibitor, 1% NP-40, 0.5% sodium deoxycholate) and 50 μl of 4× sample loading buffer. Proteins were electrophoretically separated using 10 to 12% polyacrylamide gels. Following the electrophoretic transfer of proteins onto polyvinylidene difluoride membranes (Millipore, IPVH00010), nonspecific binding was blocked by incubation with 5% skim milk in tris-buffered saline with Tween 20 (TBST). Membranes were incubated overnight at 4°C with the following primary antibodies: caspase-1 (1:1000; AdipoGen, AG-20B-0042), caspase-3 [1:1000; Cell Signaling Technology (CST), 9662S], cleaved caspase-3 (1:1000; CST, 9661S), caspase-6 (1:1000; CST, 9762S), cleaved caspase-6 (1:1000; CST, 9761S), caspase-7 (1:1000; CST, 9492S), cleaved caspase-7 (1:2000; CST, 9491S), caspase-8 (1:1000; AdipoGen, AG-20 T-0137), caspase-9 (1:1000; CST, 9508S), phosphorylated MLKL (pMLKL; 1:1000; CST, 37333S), MLKL (1:1000; AdipoGen, AP14272B), phosphorylated RIPK3 (pRIPK3; 1:1000; CST, 91702S), RIPK3 (1:1000; CST, 95702S), GSDMD (1:1000; Abcam, ab209845), GSDME (1:1000; Abcam, ab215191), ZBP1 (1:1000; AdipoGen, AG-20B-0010-C100), LDHA (1:1000; Proteintech, 19987-1-AP), HMGB1 (1:1000; Abcam, ab18256), SAPK/JNK (1:1000; CST, 9252L), phospho-SAPK/JNK (1:1000; CST, 9251L), p38 MAPK (1:1000; CST, 9212L), phospho-p38 MAPK (1:1000; CST, 4511L), ADAR1 (1:250; Santa Cruz, sc-73408), ISG15 (1:250; Santa Cruz, sc-166755), β-actin (1:1000; CST, 8457S), and glyceraldehyde phosphate dehydrogenase (GAPDH; 1:50,000; Proteintech, 60004-1-lg and 1:1000; CST, 5174S) antibodies. Membranes were then washed with TBST (10 min, three times) and incubated with the following secondary antibodies for 1 hour: horseradish peroxidase (HRP)–conjugated anti-rabbit (1:5000; Thermo Fisher Scientific, 31460) or HRP-conjugated anti-mouse (1:5000; Cellnest, CNG004-0005), followed by washing with TBST (10 min, four times). Proteins were visualized using Immobilon Forte Western HRP Substrate (Millipore, WBLUF0500), and membranes were analyzed using Amersham ImageQuant 800 ultraviolet (UV).

### Quantitative real-time PCR analysis

Extracted RNA using TRIzol reagent (GlpBio, GK20008) or RNA from immunoprecipitated samples was reversed to cDNA using the M-MLV cDNA synthesis kit (Enzynomics, EZ006S). The resulting cDNA was used as the template for quantitative PCR using SYBR Green PCR master mix (GlpBio, GK10002) on a QuantStudio 3 Real-Time PCR System (Thermo Fisher Scientific). Primers are listed in table S1.

### In vivo ethanol feeding

Age- and sex-matched, 10- to 12-week-old WT, *Ifnar1*^−/−^, *Mlkl*^−/−^, and *Zbp1*^−/−^ mice were subjected to the National Institute on Alcohol Abuse and Alcoholism model of alcoholic liver disease (*29*) adapted to include intraperitoneal IFN injections. Mice were initially acclimatized with a liquid control diet for 6 days. They were then divided into four groups: pair-fed and ethanol-fed groups with and without IFN injection. After acclimatization, the ethanol-fed groups received a liquid diet containing 5% (v/v) ethanol (Bio-Serv, F1697SP) for 10 days. The pair-fed control group received a liquid diet containing an isocaloric control (Bio-Serv, F1259SP) for 10 days. For IFN administration, mice received intraperitoneal injections of 1 μg of mouse IFN-β (PBL assay science, 12400-1) or IFN-γ (PeproTech, 315-05) on days −3, 0, 3, and 6. On day 10, pair-fed control mice received maltose dextrin by oral gavage, and the ethanol-fed mice received ethanol (5 g/kg) by oral gavage. Mice were euthanized 12 hours postgavage. The raw data values for the body weight change of WT mice are provided in table S2.

### Histopathology

Livers from mice were fixed in 10% neutral buffered formalin (Biosesang, FR2013-100-00) for hematoxylin and eosin (H&E) staining, Oil red O staining, and TUNEL assay.

### ALT and cytokine analysis

ALT and IL-1β in the serum were detected using the ALT assay kit (Elabscience, E-BC-K235-M) and enzyme-linked immunosorbent assay (Invitrogen, BMS6002TEN), respectively, according to the manufacturers’ instructions. The raw data values for these measurements are provided in tables S3 to S6.

### Dot blot analysis

Dot blot for Z-RNA analysis was performed as previously described (*38*). RNA was extracted from cells using the TRIzol reagent according to the manufacturer’s instructions and dissolved in RNase-free H_2_O. The concentration and quality of RNA were quantified using the NanoDrop UV/Vis spectrophotometer (Thermo Fisher Scientific, ND-ONE 504-108) and diluted to a final concentration of 500 ng/μl. An equal volume of RNA was directly dotted on a positively charged nylon membrane (Cytiva, RPN203B), dried, and autocrosslinked in UV Stratalinker 1800 (Stratagene, MW31) two times for 3 min. Subsequently, the membrane was blocked with 5% skim milk and then incubated with Z-NA antibody (Absolute Antibody, Ab0078-23.0). For RNase treatment, RNA samples were incubated with RNase A (1 mg/ml) at 37°C for 15 min before membrane spotting. Following incubation, the membrane was processed as described (*38*). Total double-stranded RNA was visualized using 0.2% methylene blue (Sigma-Aldrich, M9140).

### Immunofluorescence staining

Primary BMDMs were seeded onto four-chamber slides at a density of 5 × 10^5^ cells per well and incubated overnight. After stimulation, cells were washed with PBS and fixed with 4% paraformaldehyde in PBS for 15 min at room temperature, followed by five PBS washes. Cells were blocked and permeabilized in PBS containing 1% bovine serum albumin (BSA) and 0.3% Triton X-100 (Sigma-Aldrich, T8787) for 1 hour at room temperature. Samples were incubated overnight at 4°C with anti-Z-NA antibody (1:250) and anti-ZBP1 antibody (1:500) diluted in 1% BSA in PBS. For RNase treatment, RNase A (1 mg/ml) was treated at 37°C for 1 hour before primary antibody incubation. After three PBS washes, cells were incubated with Alexa Fluor 488 AffiniPure F(ab’)_2_ Fragment Donkey Anti-Mouse IgG (H + L) (1:800; Jackson ImmunoResearch, 715-546-150) and Cy3 AffiniPure Goat Anti-Rabbit IgG (H + L) (1:800; Jackson ImmunoResearch, 111-165-003) for 1 hour at room temperature. Following five PBS washes, nuclei were counterstained with 4′,6-diamidino-2-phenylindole (Invitrogen, D1306) in 1% BSA solution in PBS for 10 min at room temperature. After four PBS washes and one rinse with distilled water, samples were mounted with Immu-Mount (Epredia, 9990402). Images were acquired using the LSM 700 system (Carl Zeiss) and processed with the ZEISS ZEN 3.11 software.

### RNA immunoprecipitation

Primary BMDMs were seeded at 2 × 10^7^ cells in a 100-mm dish and stimulated. RIP was performed using the Magna RIP RNA-Binding Protein Immunoprecipitation kit (Millipore) following the manufacturer’s protocol. Cell pellets were lysed in RIP lysis buffer and incubated overnight at 4°C with magnetic beads conjugated to anti–Z-NA antibody (Absolute Antibody, Ab00783-3.0) and isotype control antibody in RIP buffer. After, bead-bound complexes were digested with proteinase K, and RNA was extracted using phenol:chloroform:isoamyl alcohol (125:24:1, pH 4.3) (Sigma-Aldrich, P1944). Purified RNA was precipitated, washed, and resuspended in nuclease-free water. The concentration and quality of RNA were quantified using the NanoDrop UV/Vis spectrophotometer.

### RNA-seq and public data analysis

To identify differentially expressed genes (DEGs) in pBMDMs compared with iBMDMs under basal conditions, RNA-seq was conducted. RNA-seq libraries were prepared from extracted RNA samples using the TruSeq Stranded mRNA Library Prep Kit (Illumina), and sequencing was performed using the Illumina sequencer in 101-bp paired-end. RNA-seq reads were aligned to the reference genome of *Mus musculus* using HISAT2 (*52*). Transcript assembly and gene expression quantification were conducted using StringTie v2.1.3b (*53*, *54*). Genes with low expression (defined by genes with an average expression ≤ 0.5 across the samples) were removed, resulting in a final set of 12,580 genes. To analyze DEGs, the “exactTest” function from edgeR v4.2.0 (*55*) in R v4.4.1 was used. Genes were considered differentially expressed if their absolute log_2_ fold change values were above 2 and *P* values were below 0.05. Hallmark pathways were downloaded as gene sets from the Molecular Signatures Database (MSigDB) (*56*); MSigDB defines these as follows: Hallmark gene sets summarize and represent specific well-defined biological states or processes, display coherent expression, and were generated by a computational approach based on identifying overlaps between gene sets in other MSigDB collections and retaining genes displaying coordinate expression (*56*). Gene set enrichment analysis (GSEA) (*57*, *58*) was performed using the fgsea v1.30.0 package (*59*) in R to determine the normalized enrichment scores (NES) for each pathway along with the significance of enrichment. The NES for all hallmark pathways was depicted together as a heatmap using the ComplexHeatmap v2.8.0 package (*60*).

To identify DEGs between healthy controls and patients with alcoholic liver disease, we obtained a bulk RNA-seq dataset from the GepLiver database (*30*) marked as GepLiver-bulk-11, comprising five healthy donors and five alcoholic hepatitis liver samples. Similarly, we obtained bulk RNA-seq data from the GepLiver database marked as GepLiver-bulk-05, comprising 26 healthy donors and 31 NAFLD, for analyzing DEGs. The DESeq2 v1.44.0 (*61*) package was used to read the count matrices and remove genes that were not expressed in at least one sample, and the limma v3.60.2 package (*61*, *62*) in R v4.4.1 was used to determine differential expression. DEGs were defined using a less stringent condition of |log fold change| > 0.5 and *P* value < 0.1. We performed hallmark pathway enrichment using the hallmark gene sets and sorted log fold change of all genes by passing them to the “fgsea” function from the fgsea package. The resulting NES activity of all pathways was showcased as a heatmap.

We performed single-sample GSEA for cell death pathways using the GSVA v1.52.2 (*63*) package. By passing the normalized count matrices along with gene sets of cell death pathways, such as apoptosis, pyroptosis, and necroptosis, obtained from MSigDB, we estimated the pathway activity matrix. The statistical significance of the difference in activity between the control versus alcoholic liver disease and control versus NAFLD samples for each pathway was performed using the Wilcoxon rank sum test (*64*).

### Statistical analysis

Unless stated otherwise, all experiments were conducted with at least three independent biological replicates. Statistical significance was assessed using a two-tailed *t* test, one-way analysis of variance (ANOVA), or two-way ANOVA, as indicated in the figure legends. All statistical analysis was performed using the GraphPad Prism 10.0 software.

## References

[1] L. A. Diaz, J. P. Arab, A. Louvet, R. Bataller, M. Arrese, The intersection between alcohol-related liver disease and nonalcoholic fatty liver disease. Nat. Rev. Gastroenterol. Hepatol. 20, 764–783 (2023). 37582985 10.1038/s41575-023-00822-y

[2] H. K. Seitz, F. Stickel, Molecular mechanisms of alcohol-mediated carcinogenesis. Nat. Rev. Cancer 7, 599–612 (2007). 17646865 10.1038/nrc2191

[3] D. Q. Huang, P. Mathurin, H. Cortez-Pinto, R. Loomba, Global epidemiology of alcohol-associated cirrhosis and HCC: Trends, projections and risk factors. Nat. Rev. Gastroenterol. Hepatol. 20, 37–49 (2023). 36258033 10.1038/s41575-022-00688-6 PMC9579565

[4] J. Fernandez-Sola, Cardiovascular risks and benefits of moderate and heavy alcohol consumption. Nat. Rev. Cardiol. 12, 576–587 (2015). 26099843 10.1038/nrcardio.2015.91

[5] K. Witkiewitz, R. Z. Litten, L. Leggio, Advances in the science and treatment of alcohol use disorder. Sci. Adv. 5, eaax4043 (2019). 31579824 10.1126/sciadv.aax4043 PMC6760932

[6] P. K. Im, N. Wright, L. Yang, K. H. Chan, Y. Chen, Y. Guo, H. Du, X. Yang, D. Avery, S. Wang, C. Yu, J. Lv, R. Clarke, J. Chen, R. Collins, R. G. Walters, R. Peto, L. Li, Z. Chen, I. Y. Millwood, China Kadoorie Biobank Collaborative Group, Alcohol consumption and risks of more than 200 diseases in Chinese men. Nat. Med. 29, 1476–1486 (2023). 37291211 10.1038/s41591-023-02383-8 PMC10287564

[7] S. K. Ramaiah, H. Jaeschke, Hepatic neutrophil infiltration in the pathogenesis of alcohol-induced liver injury. Toxicol. Mech. Methods 17, 431–440 (2007). 20020946 10.1080/00952990701407702

[8] A. Bertola, O. Park, B. Gao, Chronic plus binge ethanol feeding synergistically induces neutrophil infiltration and liver injury in mice: A critical role for E-selectin. Hepatology 58, 1814–1823 (2013). 23532958 10.1002/hep.26419 PMC3726575

[9] H. J. Wang, B. Gao, S. Zakhari, L. E. Nagy, Inflammation in alcoholic liver disease. Annu. Rev. Nutr. 32, 343–368 (2012). 22524187 10.1146/annurev-nutr-072610-145138 PMC3670145

[10] C. Brechot, B. Nalpas, A. M. Courouce, G. Duhamel, P. Callard, F. Carnot, P. Tiollais, P. Berthelot, Evidence that hepatitis B virus has a role in liver-cell carcinoma in alcoholic liver disease. N. Engl. J. Med. 306, 1384–1387 (1982). 6281640 10.1056/NEJM198206103062302

[11] B. Gao, Interaction of alcohol and hepatitis viral proteins: Implication in synergistic effect of alcohol drinking and viral hepatitis on liver injury. Alcohol 27, 69–72 (2002). 12062640 10.1016/s0741-8329(02)00201-x

[12] T. Marjot, A. M. Moon, J. A. Cook, S. Abd-Elsalam, C. Aloman, M. J. Armstrong, E. Pose, E. J. Brenner, T. Cargill, M. A. Catana, R. Dhanasekaran, A. Eshraghian, I. Garcia-Juarez, U. S. Gill, P. D. Jones, J. Kennedy, A. Marshall, C. Matthews, G. Mells, C. Mercer, P. V. Perumalswami, E. Avitabile, X. Qi, F. Su, N. N. Ufere, Y. J. Wong, M. H. Zheng, E. Barnes, A. S. Barritt IV, G. J. Webb, Outcomes following SARS-CoV-2 infection in patients with chronic liver disease: An international registry study. J. Hepatol. 74, 567–577 (2021). 33035628 10.1016/j.jhep.2020.09.024 PMC7536538

[13] S. M. Man, T. D. Kanneganti, Innate immune sensing of cell death in disease and therapeutics. Nat. Cell Biol. 26, 1420–1433 (2024). 39223376 10.1038/s41556-024-01491-y PMC12459733

[14] H. Kwak, E. Lee, R. Karki, DNA sensors in metabolic and cardiovascular diseases: Molecular mechanisms and therapeutic prospects. Immunol. Rev. 329, e13382 (2025). 39158380 10.1111/imr.13382 PMC11744256

[15] S. M. Man, B. J. Jenkins, Context-dependent functions of pattern recognition receptors in cancer. Nat. Rev. Cancer 22, 397–413 (2022). 35355007 10.1038/s41568-022-00462-5

[16] T. Miyata, L. E. Nagy, Programmed cell death in alcohol-associated liver disease. Clin. Mol. Hepatol. 26, 618–625 (2020). 32951412 10.3350/cmh.2020.0142 PMC7641549

[17] A. L. King, T. M. Swain, Z. Mao, U. S. Udoh, C. R. Oliva, A. M. Betancourt, C. E. Griguer, D. R. Crowe, M. Lesort, S. M. Bailey, Involvement of the mitochondrial permeability transition pore in chronic ethanol-mediated liver injury in mice. Am. J. Physiol. Gastrointest. Liver Physiol. 306, G265–G277 (2014). 24356880 10.1152/ajpgi.00278.2013 PMC3920122

[18] S. Wang, P. Pacher, R. C. De Lisle, H. Huang, W. X. Ding, A mechanistic review of cell death in alcohol-induced liver injury. Alcohol. Clin. Exp. Res. 40, 1215–1223 (2016). 27130888 10.1111/acer.13078 PMC5455778

[19] S. Roychowdhury, M. R. McMullen, S. G. Pisano, X. Liu, L. E. Nagy, Absence of receptor interacting protein kinase 3 prevents ethanol-induced liver injury. Hepatology 57, 1773–1783 (2013). 23319235 10.1002/hep.26200 PMC3628968

[20] T. Miyata, X. Wu, X. Fan, E. Huang, C. Sanz-Garcia, C. K. C. Ross, S. Roychowdhury, A. Bellar, M. R. McMullen, J. Dasarathy, D. S. Allende, J. Caballeria, P. Sancho-Bru, C. J. McClain, M. Mitchell, A. J. McCullough, S. Radaeva, B. Barton, G. Szabo, S. Dasarathy, L. E. Nagy, Differential role of MLKL in alcohol-associated and non-alcohol-associated fatty liver diseases in mice and humans. JCI Insight 6, e140180 (2021). 33616081 10.1172/jci.insight.140180 PMC7934930

[21] A. H. Lau, G. Szabo, A. W. Thomson, Antigen-presenting cells under the influence of alcohol. Trends Immunol. 30, 13–22 (2009). 19059005 10.1016/j.it.2008.09.005

[22] X.-Y. Song, P.-C. Liu, W.-W. Liu, J. Zhou, T. Hayashi, K. Mizuno, S. Hattori, H. Fujisaki, T. Ikejima, Silibinin inhibits ethanol- or acetaldehyde-induced ferroptosis in liver cell lines. Toxicol. In Vitro 82, 105388 (2022). 35595033 10.1016/j.tiv.2022.105388

[23] I. Hritz, P. Mandrekar, A. Velayudham, D. Catalano, A. Dolganiuc, K. Kodys, E. Kurt-Jones, G. Szabo, The critical role of toll-like receptor (TLR) 4 in alcoholic liver disease is independent of the common TLR adapter MyD88. Hepatology 48, 1224–1231 (2008). 18792393 10.1002/hep.22470 PMC7137387

[24] J. Petrasek, S. Bala, T. Csak, D. Lippai, K. Kodys, V. Menashy, M. Barrieau, S. Y. Min, E. A. Kurt-Jones, G. Szabo, IL-1 receptor antagonist ameliorates inflammasome-dependent alcoholic steatohepatitis in mice. J. Clin. Invest. 122, 3476–3489 (2012). 22945633 10.1172/JCI60777 PMC3461900

[25] Y. Xie, Z. Wang, G. Song, H. Ma, B. Feng, GSDMD induces hepatocyte pyroptosis to trigger alcoholic hepatitis through modulating mitochondrial dysfunction. Cell Div. 19, 10 (2024). 38532477 10.1186/s13008-024-00114-0 PMC10964551

[26] R. Sartorius, M. Trovato, R. Manco, L. D'Apice, P. De Berardinis, Exploiting viral sensing mediated by Toll-like receptors to design innovative vaccines. NPJ Vaccines 6, 127 (2021). 34711839 10.1038/s41541-021-00391-8 PMC8553822

[27] M. A. Campos, G. P. Zolini, E. G. Kroon, Impact of Toll-Like Receptors (TLRs) and TLR signaling proteins in trigeminal ganglia impairing herpes simplex virus 1 (HSV-1) progression to encephalitis: Insights from mouse models. Front. Biosci. (Landmark Ed) 29, 102 (2024). 38538263 10.31083/j.fbl2903102

[28] J. Luo, G. Song, N. Chen, M. Xie, X. Niu, S. Zhou, Y. Ji, X. Zhu, W. Ma, Q. Zhang, D. Yu, Ferroptosis contributes to ethanol-induced hepatic cell death via labile iron accumulation and GPx4 inactivation. Cell Death Discov. 9, 311 (2023). 37626043 10.1038/s41420-023-01608-6 PMC10457354

[29] A. Bertola, S. Mathews, S. H. Ki, H. Wang, B. Gao, Mouse model of chronic and binge ethanol feeding (the NIAAA model). Nat. Protoc. 8, 627–637 (2013). 23449255 10.1038/nprot.2013.032 PMC3788579

[30] Z. Li, H. Zhang, Q. Li, W. Feng, X. Jia, R. Zhou, Y. Huang, Y. Li, Z. Hu, X. Hu, X. Zhu, S. Huang, GepLiver: An integrative liver expression atlas spanning developmental stages and liver disease phases. Sci. Data 10, 376 (2023). 37301898 10.1038/s41597-023-02257-1 PMC10257690

[31] E. Lee, C. H. Song, S. J. Bae, K. T. Ha, R. Karki, Regulated cell death pathways and their roles in homeostasis, infection, inflammation, and tumorigenesis. Exp. Mol. Med. 55, 1632–1643 (2023). 37612410 10.1038/s12276-023-01069-y PMC10474065

[32] S. R. Dubey, C. Turnbull, A. Pandey, A. Zhao, M. Kurera, R. Al-Zidan, C. Shen, M. Gautam, S. Mahajan, P. S. Jadhav, A. Ghosh, C. Ngo, S. M. Man, Molecular mechanisms and regulation of inflammasome activation and signaling: Sensing of pathogens and damage molecular patterns. Cell. Mol. Immunol. 22, 1313–1344 (2025). 41062723 10.1038/s41423-025-01354-y PMC12575685

[33] S. Lee, R. Karki, Y. Wang, L. N. Nguyen, R. C. Kalathur, T. D. Kanneganti, AIM2 forms a complex with pyrin and ZBP1 to drive PANoptosis and host defence. Nature 597, 415–419 (2021). 34471287 10.1038/s41586-021-03875-8 PMC8603942

[34] K. Kelepouras, J. Saggau, D. Bonasera, C. Kiefer, F. Locci, H. Rakhsh-Khorshid, L. Grauvogel, A. B. Varanda, M. Peifer, E. Loricchio, A. Montinaro, M. Croon, A. Trifunovic, G. Prencipe, A. Insalaco, F. De Benedetti, H. Walczak, G. Liccardi, STING induces ZBP1-mediated necroptosis independently of TNFR1 and FADD. Nature 647, 735–746 (2025). 40834903 10.1038/s41586-025-09536-4 PMC12629989

[35] R. Karki, B. Sundaram, B. R. Sharma, S. Lee, R. K. S. Malireddi, L. N. Nguyen, S. Christgen, M. Zheng, Y. Wang, P. Samir, G. Neale, P. Vogel, T. D. Kanneganti, ADAR1 restricts ZBP1-mediated immune response and PANoptosis to promote tumorigenesis. Cell Rep. 37, 109858 (2021). 34686350 10.1016/j.celrep.2021.109858 PMC8853634

[36] J. Maelfait, J. Rehwinkel, The Z-nucleic acid sensor ZBP1 in health and disease. J. Exp. Med. 220, e20221156 (2023). 37450010 10.1084/jem.20221156 PMC10347765

[37] H. Bae, S. Moon, M. Chang, F. Zhang, Y. Jang, W. Kim, S. Kim, M. Fu, J. Lim, S. Park, C. N. Patel, R. Mall, M. Zheng, S. M. Man, R. Karki, Ferroptosis-activating metabolite acrolein antagonizes necroptosis and anti-cancer therapeutics. Nat. Commun. 16, 4919 (2025). 40425585 10.1038/s41467-025-60226-1 PMC12116918

[38] T. Yang, G. Wang, M. Zhang, X. Hu, Q. Li, F. Yun, Y. Xing, X. Song, H. Zhang, G. Hu, Y. Qian, Triggering endogenous Z-RNA sensing for anti-tumor therapy through ZBP1-dependent necroptosis. Cell Rep. 42, 113377 (2023). 37922310 10.1016/j.celrep.2023.113377

[39] T. Zhang, C. Yin, A. Fedorov, L. Qiao, H. Bao, N. Beknazarov, S. Wang, A. Gautam, R. M. Williams, J. C. Crawford, S. Peri, V. Studitsky, A. A. Beg, P. G. Thomas, C. Walkley, Y. Xu, M. Poptsova, A. Herbert, S. Balachandran, ADAR1 masks the cancer immunotherapeutic promise of ZBP1-driven necroptosis. Nature 606, 594–602 (2022). 35614224 10.1038/s41586-022-04753-7 PMC9373927

[40] K. Hattori, I. Naguro, C. Runchel, H. Ichijo, The roles of ASK family proteins in stress responses and diseases. Cell Commun. Signal 7, 9 (2009). 19389260 10.1186/1478-811X-7-9 PMC2685135

[41] G. H. Tesch, F. Y. Ma, D. J. Nikolic-Paterson, Targeting apoptosis signal-regulating kinase 1 in acute and chronic kidney disease. Anat. Rec. (Hoboken) 303, 2553–2560 (2020). 31971352 10.1002/ar.24373

[42] J. Zheng, Q. Dai, K. Han, W. Hong, D. Jia, Y. Mo, Y. Lv, H. Tang, H. Fu, W. Geng, JNK-IN-8, a c-Jun N-terminal kinase inhibitor, improves functional recovery through suppressing neuroinflammation in ischemic stroke. J. Cell. Physiol. 235, 2792–2799 (2020). 31541462 10.1002/jcp.29183 PMC6916328

[43] T. Kuriakose, S. M. Man, R. K. Malireddi, R. Karki, S. Kesavardhana, D. E. Place, G. Neale, P. Vogel, T.-D. Kanneganti, ZBP1/DAI is an innate sensor of influenza virus triggering the NLRP3 inflammasome and programmed cell death pathways. Sci. Immunol. 1, aag2045 (2016). 27917412 10.1126/sciimmunol.aag2045 PMC5131924

[44] R. Karki, S. Lee, R. Mall, N. Pandian, Y. Wang, B. R. Sharma, R. S. Malireddi, D. Yang, S. Trifkovic, J. A. Steele, J. P. Connelly, G. Vishwanath, M. Sasikala, D. N. Reddy, P. Vogel, S. M. Pruett-Miller, R. Webby, C. B. Jonsson, T.-D. Kanneganti, ZBP1-dependent inflammatory cell death, PANoptosis, and cytokine storm disrupt IFN therapeutic efficacy during coronavirus infection. Sci. Immunol. 7, eabo6294 (2022). 35587515 10.1126/sciimmunol.abo6294 PMC9161373

[45] R. Karki, T. D. Kanneganti, ADAR1 and ZBP1 in innate immunity, cell death, and disease. Trends Immunol. 44, 201–216 (2023). 36710220 10.1016/j.it.2023.01.001 PMC9974732

[46] S. Oh, J. Lee, J. Oh, G. Yu, H. Ryu, D. Kim, S. Lee, Integrated NLRP3, AIM2, NLRC4, Pyrin inflammasome activation and assembly drive PANoptosis. Cell. Mol. Immunol. 20, 1513–1526 (2023). 38008850 10.1038/s41423-023-01107-9 PMC10687226

[47] J.-H. Han, R. Karki, R. K. S. Malireddi, R. Mall, R. Sarkar, B. R. Sharma, J. Klein, H. Berns, H. Pisharath, S. M. Pruett-Miller, S.-J. Bae, T.-D. Kanneganti, NINJ1 mediates inflammatory cell death, PANoptosis, and lethality during infection conditions and heat stress. Nat. Commun. 15, 1739 (2024). 38409108 10.1038/s41467-024-45466-x PMC10897308

[48] E. Blasi, D. Radzioch, L. Merletti, L. Varesio, Generation of macrophage cell line from fresh bone marrow cells with a myc/raf recombinant retrovirus. Cancer Biochem. Biophys. 10, 303–317 (1989). 2695237

[49] M. Charni-Natan, I. Goldstein, Protocol for primary mouse hepatocyte isolation. STAR Protoc. 1, 100086 (2020). 33111119 10.1016/j.xpro.2020.100086 PMC7580103

[50] W. Kim, S. Kim, H. Woo, R. A. Jojare, R. Mall, A. Nicotra, B. F. Py, C. Ngo, S. M. Man, C. N. Patel, R. Karki, A potent NLRP3 inhibitor effective against both MCC950-sensitive and -resistant inflammation. Cell Chem. Biol. 32, 1125–1139.e7 (2025). 40930090 10.1016/j.chembiol.2025.08.006

[51] R. Karki, S. M. Man, R. K. S. Malireddi, P. Gurung, P. Vogel, M. Lamkanfi, T. D. Kanneganti, Concerted activation of the AIM2 and NLRP3 inflammasomes orchestrates host protection against Aspergillus infection. Cell Host Microbe 17, 357–368 (2015). 25704009 10.1016/j.chom.2015.01.006 PMC4359672

[52] D. Kim, B. Langmead, S. L. Salzberg, HISAT: A fast spliced aligner with low memory requirements. Nat. Methods 12, 357–360 (2015). 25751142 10.1038/nmeth.3317 PMC4655817

[53] M. Pertea, G. M. Pertea, C. M. Antonescu, T. C. Chang, J. T. Mendell, S. L. Salzberg, StringTie enables improved reconstruction of a transcriptome from RNA-seq reads. Nat. Biotechnol. 33, 290–295 (2015). 25690850 10.1038/nbt.3122 PMC4643835

[54] M. Pertea, D. Kim, G. M. Pertea, J. T. Leek, S. L. Salzberg, Transcript-level expression analysis of RNA-seq experiments with HISAT, StringTie and Ballgown. Nat. Protoc. 11, 1650–1667 (2016). 27560171 10.1038/nprot.2016.095 PMC5032908

[55] M. D. Robinson, D. J. McCarthy, G. K. Smyth, edgeR: A Bioconductor package for differential expression analysis of digital gene expression data. Bioinformatics 26, 139–140 (2010). 19910308 10.1093/bioinformatics/btp616 PMC2796818

[56] A. Liberzon, C. Birger, H. Thorvaldsdottir, M. Ghandi, J. P. Mesirov, P. Tamayo, The Molecular Signatures Database (MSigDB) hallmark gene set collection. Cell Syst. 1, 417–425 (2015). 26771021 10.1016/j.cels.2015.12.004 PMC4707969

[57] V. K. Mootha, J. Bunkenborg, J. V. Olsen, M. Hjerrild, J. R. Wisniewski, E. Stahl, M. S. Bolouri, H. N. Ray, S. Sihag, M. Kamal, N. Patterson, E. S. Lander, M. Mann, Integrated analysis of protein composition, tissue diversity, and gene regulation in mouse mitochondria. Cell 115, 629–640 (2003). 14651853 10.1016/s0092-8674(03)00926-7

[58] A. Subramanian, P. Tamayo, V. K. Mootha, S. Mukherjee, B. L. Ebert, M. A. Gillette, A. Paulovich, S. L. Pomeroy, T. R. Golub, E. S. Lander, J. P. Mesirov, Gene set enrichment analysis: A knowledge-based approach for interpreting genome-wide expression profiles. Proc. Natl. Acad. Sci. U.S.A. 102, 15545–15550 (2005). 16199517 10.1073/pnas.0506580102 PMC1239896

[59] A. A. Sergushichev, An algorithm for fast preranked gene set enrichment analysis using cumulative statistic calculation. bioRxiv 060012 [Preprint] (2016). 10.1101/060012.

[60] Z. Gu, Complex heatmap visualization. Imeta 1, e43 (2022). 38868715 10.1002/imt2.43 PMC10989952

[61] M. I. Love, W. Huber, S. Anders, Moderated estimation of fold change and dispersion for RNA-seq data with DESeq2. Genome Biol. 15, 550 (2014). 25516281 10.1186/s13059-014-0550-8 PMC4302049

[62] M. E. Ritchie, B. Phipson, D. Wu, Y. Hu, C. W. Law, W. Shi, G. K. Smyth, limma powers differential expression analyses for RNA-sequencing and microarray studies. Nucleic Acids Res. 43, e47 (2015). 25605792 10.1093/nar/gkv007 PMC4402510

[63] S. Hänzelmann, R. Castelo, J. Guinney, GSVA: Gene set variation analysis for microarray and RNA-seq data. BMC Bioinformatics 14, 7 (2013). 23323831 10.1186/1471-2105-14-7 PMC3618321

[64] W. J. Conover, *Practical Nonparametric Statistics*. (John Wiley & Sons, 1980).

