# Supplementary material for: Innate immune sensing of dietary alcohol ignites inflammation to drive alcohol-related disease
Source: Sci Adv. 2026 Apr 10;12(15):eaea3979. doi: 10.1126/sciadv.aea3979 (PMC13068059; doi:10.1126/sciadv.aea3979)
Supplement: Supplementary file 1 — Figs. S1 to S8 Tables S1 to S6 Legend for data S1 [file sciadv.aea3979_sm.pdf]

Science Advances
AAAS

Supplementary Materials for

Innate immune sensing of dietary alcohol ignites inflammation to drive alcohol-related disease

Yeonseo Jang *et al.*

Corresponding author: SangJoon Lee, sangjoon.lee@unist.ac.kr; Si Ming Man, siming.man@anu.edu.au; Rajendra Karki, rkarki@snu.ac.kr

Sci. Adv. **12**, eaea3979 (2026)
DOI: 10.1126/sciadv.aea3979

The PDF file includes:

Figs. S1 to S8
Tables S1 to S6
Legend for data S1

Other Supplementary Material for this manuscript includes the following:

Data S1

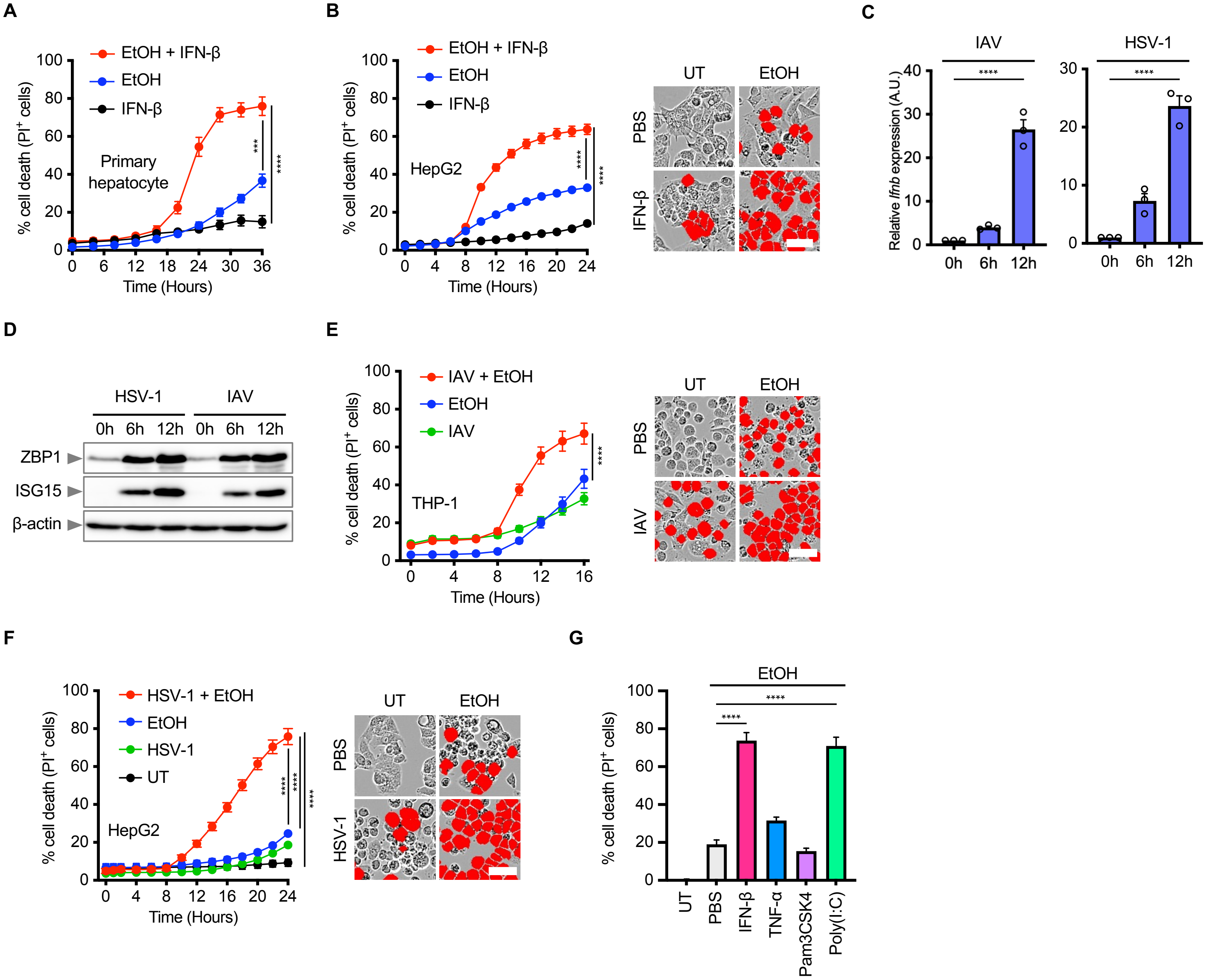

Fig. S1. Alcohol and interferon induce cell death in mouse and human cells. (**A** and **B**) Real-time analysis of cell death in (A) primary hepatocytes and (B) HepG2 cells following stimulation with 0.6 M ethanol (EtOH), 20 ng/mL IFN-β, or a combination of both which IFN-β was added 12 h prior to EtOH. Representative images of cell death (red color) of HepG2 cells are shown (right) at 16 h of EtOH treatment. Scale bar, 50 µm. (**C**) Representative quantitative real-time PCR analysis of *Ifnb* in wild-type (WT) primary bone marrow-derived macrophages (pBMDMs) infected with IAV or HSV-1. *18S rRNA* was used as an internal control. (**D**) Immunoblot analysis of ZBP1 and ISG15 in WT pBMDMs infected with HSV-1 or IAV. β-actin was used as an internal control. (**E** and **F**) Real-time analysis of cell death by 0.6 M EtOH in (E) THP-1 left uninfected or infected with IAV and (F) HepG2 cells left uninfected or infected with HSV-1. Representative images of cell death (red color) after 16 h of EtOH treatment are shown (right). Scale bars, 50 µm. (**G**) Percentage of cell death in WT pBMDMs treated with EtOH alone or in combination with IFN-β, TNF-α, Pam3CSK4, or Poly(I:C) after 3 h of EtOH treatment. Cells were pretreated with each cytokine or ligand for 12 h prior to EtOH. Data are representative of at least two independent experiments. $***p < 0.001$; $****p < 0.0001$. Analysis was performed using two-way ANOVA (A, B, E, and F) and one-way ANOVA (C and G). Data are shown as mean ± SEM (A, B, C, E, F, and G).

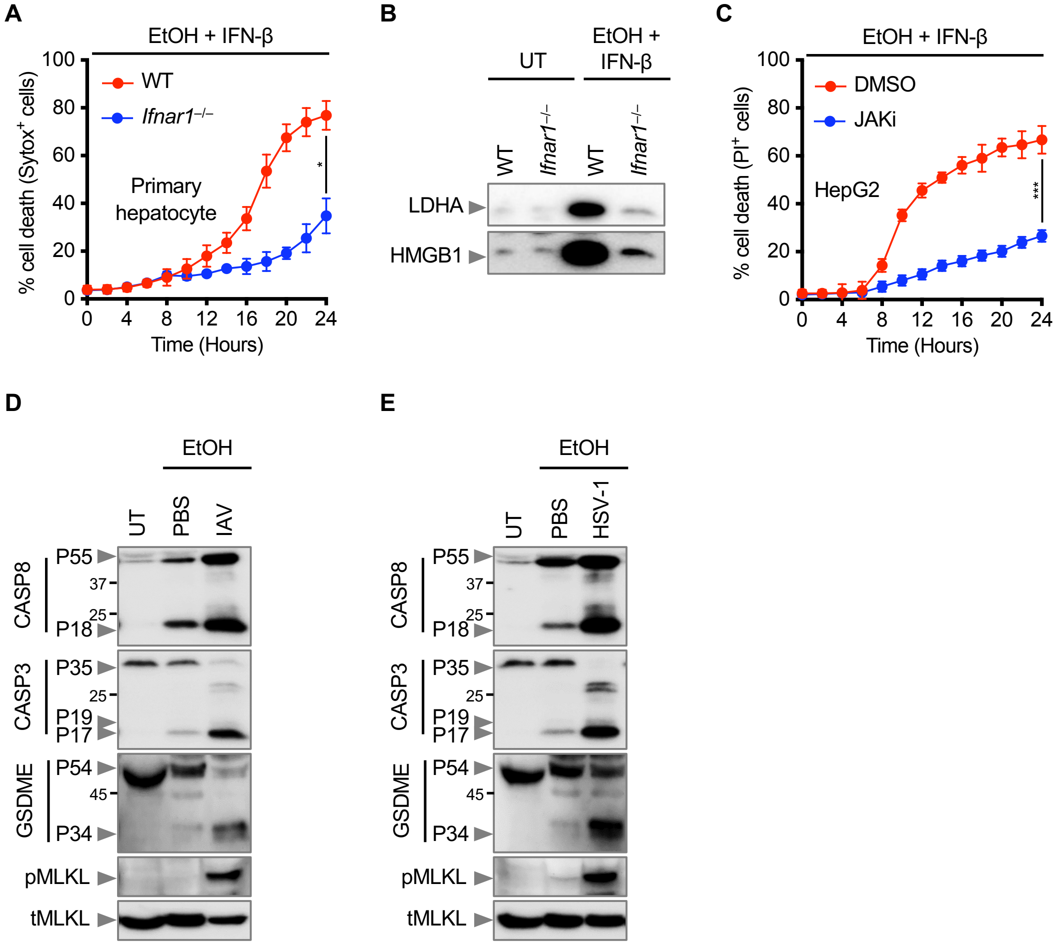

Fig. S2. Alcohol and infection-induced interferon exacerbate pyroptosis, apoptosis, and necroptosis. (**A**) Real-time analysis of cell death in wild-type (WT) and *Ifnar1*$^{-/-}$ primary hepatocytes stimulated with 0.6 M ethanol (EtOH) plus 20 ng/mL IFN-β. (**B**) Immunoblot analysis of LDHA and HMGB1 in WT and *Ifnar1*$^{-/-}$ primary bone marrow-derived macrophages (pBMDMs) stimulated with EtOH plus IFN-β. (**C**) Real-time analysis of cell death in Baricitinib (JAKi)-treated HepG2 cells stimulated with EtOH plus IFN-β. (**D** and **E**) Immunoblot analysis pro- (P55) and cleaved (P18) CASP8, pro- (P35) and cleaved (P19 and P17) CASP3, pro- (P54) and activated (P34) GSDME, phosphorylated MLKL (pMLKL), and total MLKL (tMLKL) in WT pBMDMs stimulated with 0.6 M EtOH in the presence of (D) IAV or (E) HSV-1 for 9 h. Data are representative of at least three independent experiments. *$p < 0.05$; ***$p < 0.001$. Analysis was performed using a *t*-test (A and C). Data are shown as mean ± SEM (A and C).

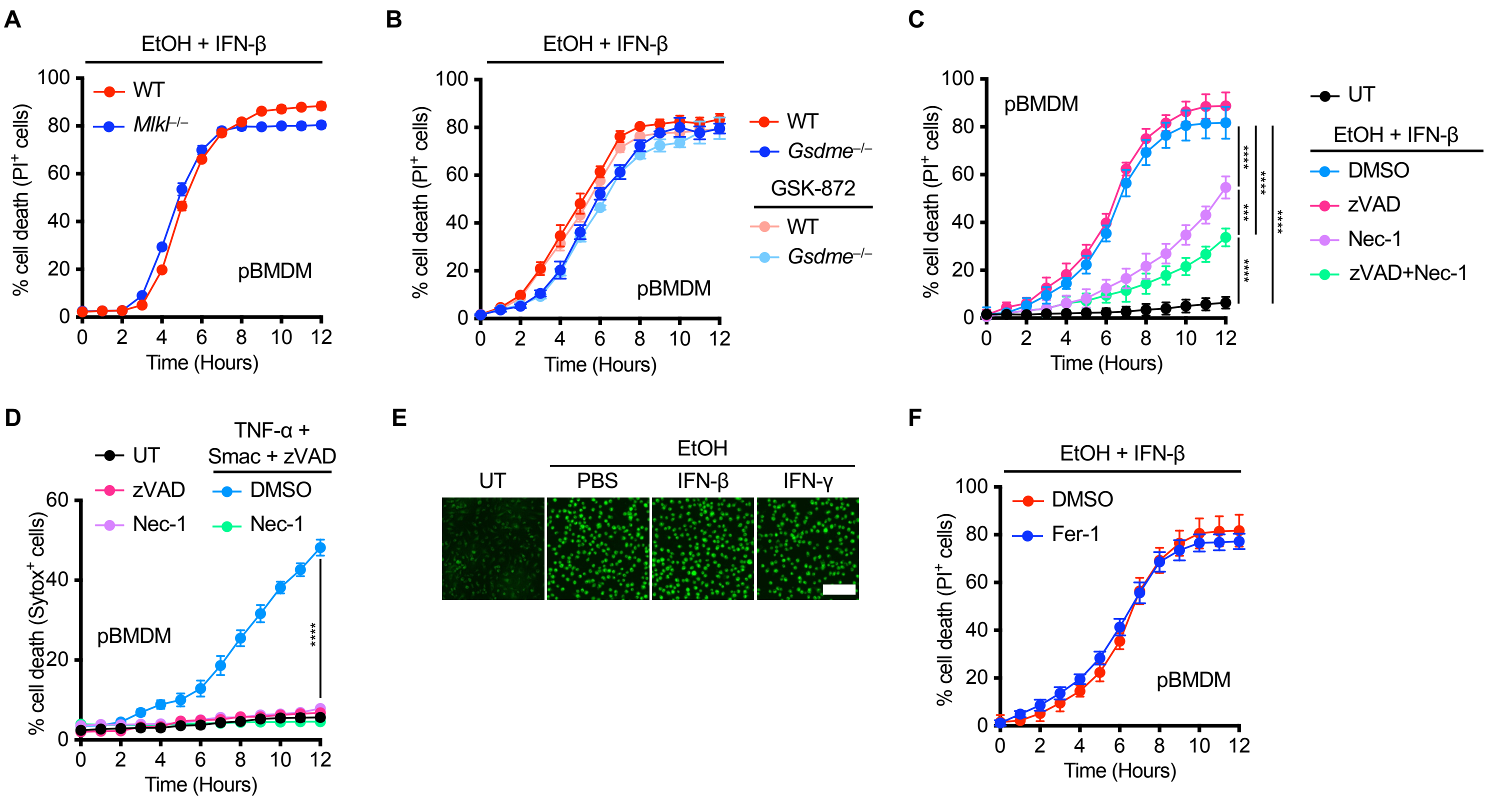

Fig. S3. Alcohol and interferon induce multiple cell death pathways cooperatively. (**A**) Real-time analysis of cell death in wild-type (WT) and *Mlkl*$^{-/-}$ primary bone marrow-derived macrophages (pBMDMs) following stimulation with 0.6 M ethanol (EtOH) plus 20 ng/mL IFN-β. (**B**) Real-time analysis of cell death in WT and *Gsdme*$^{-/-}$ pBMDMs stimulated with EtOH plus IFN-β with or without GSK-872. (**C**) Real-time analysis of cell death in WT pBMDMs stimulated with EtOH plus IFN-β in the absence or presence of the indicated cell death inhibitors. (**D**) Real-time analysis of cell death in WT pBMDMs treated as indicated. (**E**) Representative images of lipid peroxidation using BODIPY 581/591 C11 staining (green color) in WT pBMDMs stimulated with EtOH for 6 h in the presence of PBS, IFN-β, or IFN-γ. Scale bar, 100 μm. (**F**) Real-time analysis of cell death in WT pBMDMs stimulated with EtOH plus IFN-β in the presence of DMSO or Ferrostatin-1 (Fer-1). Data are representative of at least three independent experiments. ***$p < 0.001$; ****$p < 0.0001$. Analysis was performed using one-way ANOVA (A, B, and F) and two-way ANOVA (C and D). Data are shown as mean ± SEM (A, B, C, D, and F).

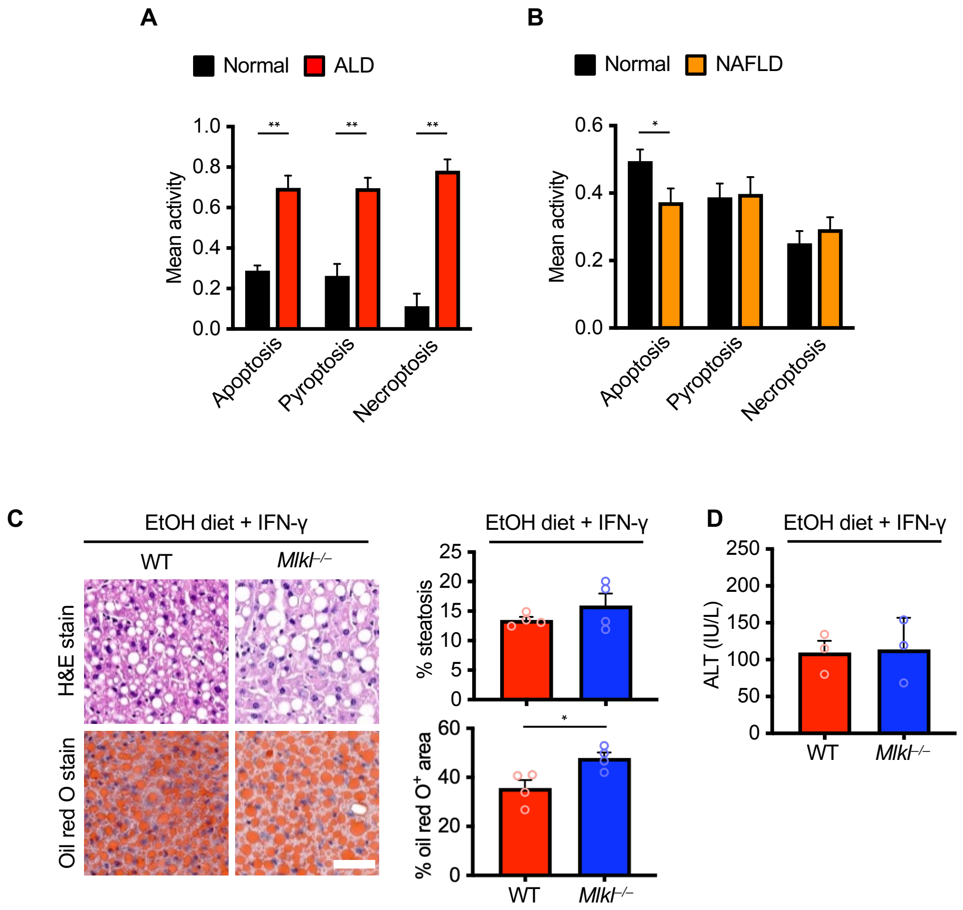

Fig. S4. Activation of multiple lytic cell death pathways in alcoholic liver disease patients and necroptosis-independent ethanol-induced liver injury in mice. (**A** and **B**) Activity of cell death pathways of apoptosis, pyroptosis, and necroptosis in (A) patients with alcoholic liver disease (ALD) compared to healthy people (normal) and (B) patients with non-alcoholic fatty liver disease (NAFLD) compared to healthy people (normal). (**C**) Histopathological analysis, H&E and Oil red O stain of liver tissues from wild-type (WT) and *Mlkl*$^{-/-}$ mice fed with ethanol (EtOH) and injected with IFN-γ. Scale bar, 200 μm. Quantifications of histopathological analyses are shown (right). (**D**) Analysis of serum ALT in WT (n=3) and *Mlkl*$^{-/-}$ (n=3) mice fed with EtOH and injected with IFN-γ. *$p < 0.05$; **$p < 0.01$. Analysis was performed using a *t*-test. Data are shown as mean ± SEM.

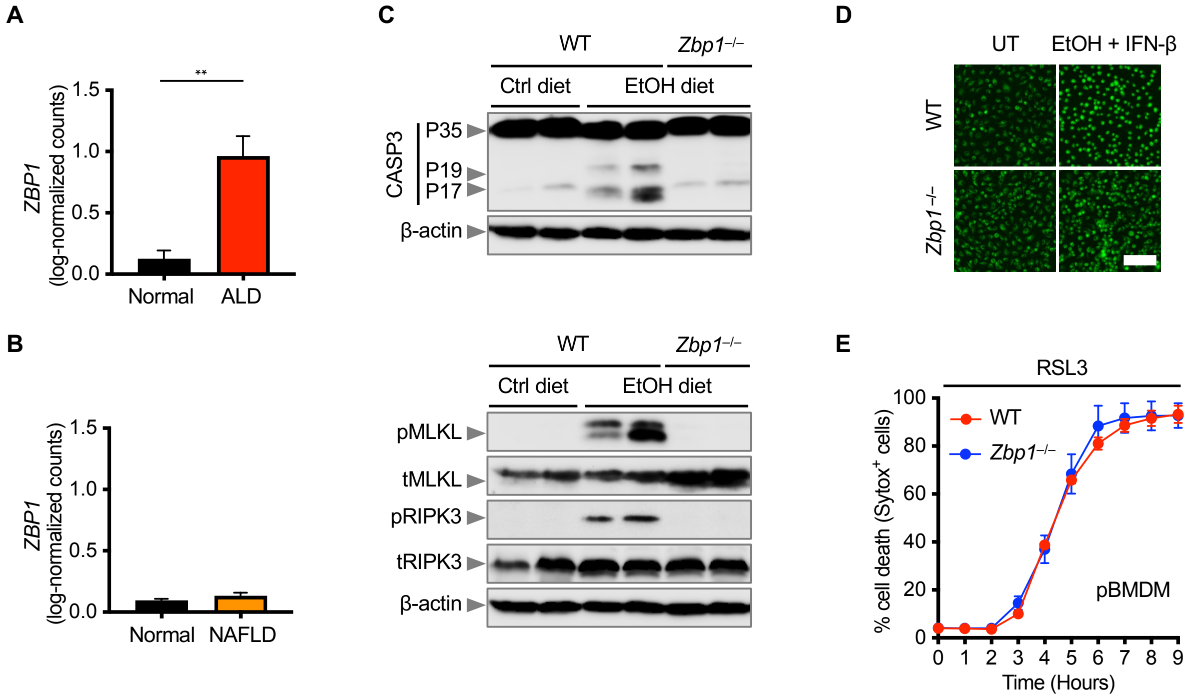

Fig. S5. ZBP1 is upregulated in alcoholic liver disease patients and activates alcohol-induced cell death in the liver. (**A** and **B**) Gene expression analysis of *ZBP1* in patients with (A) alcoholic liver disease (ALD) and (B) non-alcoholic fatty liver disease (NAFLD) relative to the expression in healthy subjects (normal). (**C**) Immunoblot analysis of pro- (P35) and cleaved (P19 and P17) CASP3, phosphorylated MLKL (pMLKL), total MLKL (tMLKL), phosphorylated RIPK3 (pRIPK3), and total RIPK3 (tRIPK3) in the liver of wild-type (WT) and *Zbp1*$^{-/-}$ mice with control (Ctrl) diet or ethanol (EtOH) diet. β-actin was used as an internal control. (**D**) Representative images of lipid peroxidation using BODIPY 581/591 C11 staining (green color) in WT and *Zbp1*$^{-/-}$ primary bone marrow-derived macrophages (pBMDMs) stimulated with 0.6 M EtOH plus 20 ng/mL IFN-β for 6 h. Scale bar, 100 µm. (**E**) Real-time analysis of cell death in WT and *Zbp1*$^{-/-}$ pBMDMs stimulated with 1 µM RSL3. $^{**}p < 0.01$. Analysis was performed using *t*-test (A and B) and one-way ANOVA (E). Data are shown as mean ± SEM (A, B, and E).

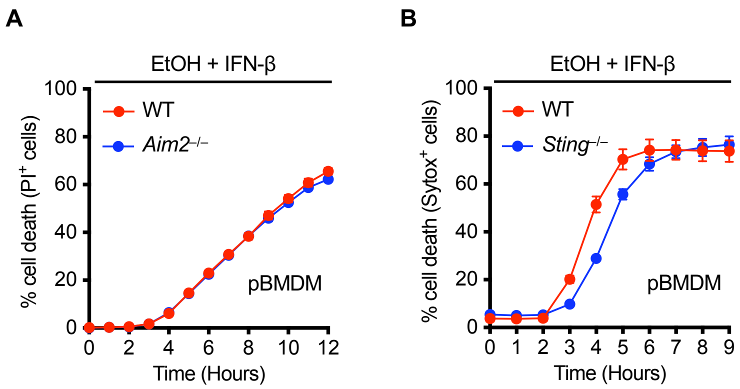

Fig. S6. AIM2 and STING are dispensable for the cell death induced by ethanol and interferon. (**A** and **B**) Real-time analysis of cell death in (A) wild-type (WT) and *Aim2*$^{-/-}$ and (B) WT and *Sting*$^{-/-}$ primary bone marrow-derived macrophages (pBMDMs) stimulated with 0.6 M ethanol (EtOH) plus 20 ng/mL IFN-β. Analysis was performed using one-way ANOVA. Data are representative of at least three experiments and shown as mean ± SEM.

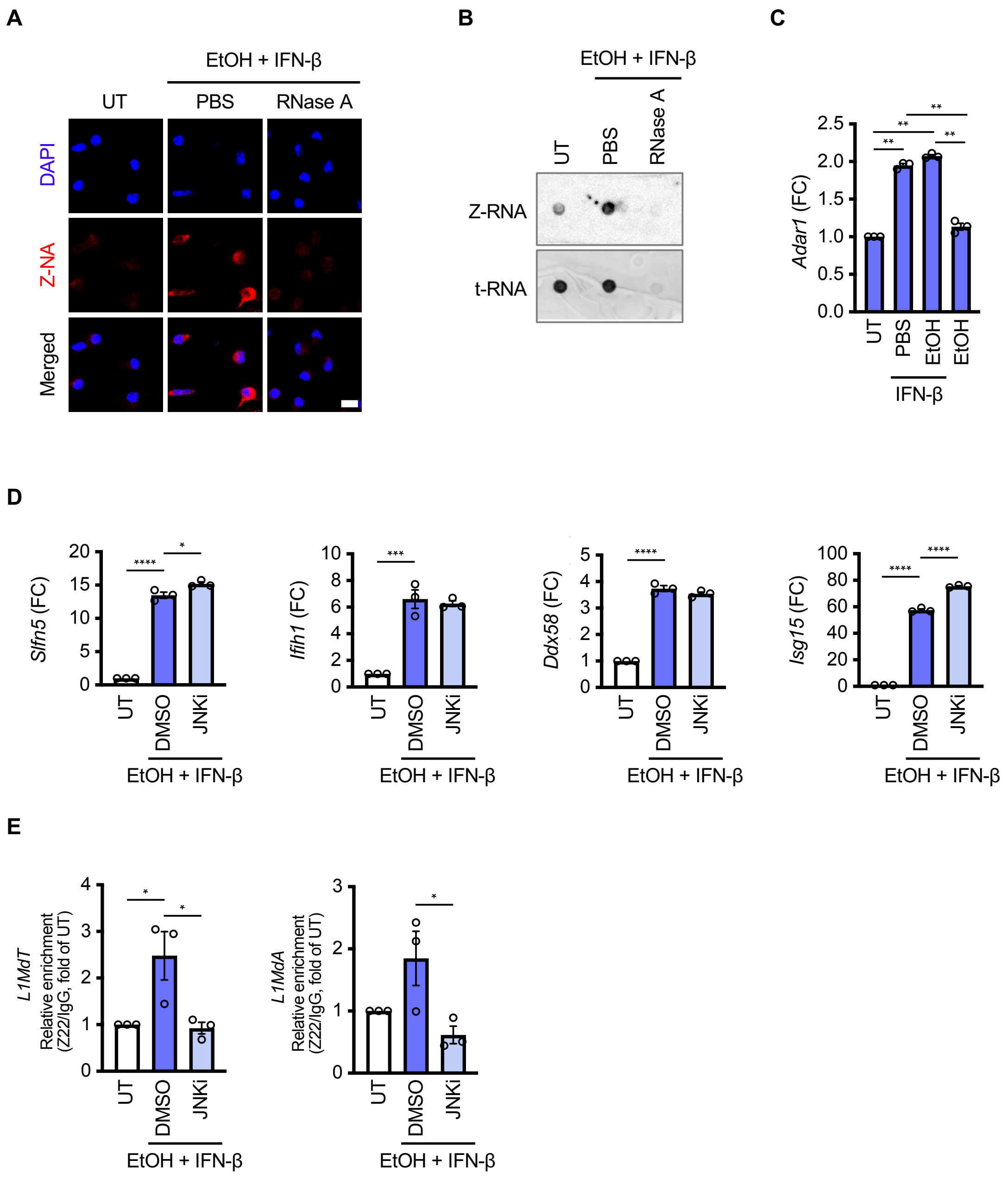

Fig. S7. JNK signaling promotes the expression of transcripts adopting Z-RNA formation. (A) Immunofluorescence images of wild-type (WT) primary bone marrow-derived macrophages (pBMDMs) treated with 0.6 M ethanol (EtOH) plus 20 ng/mL IFN-β for 3 h, with or without RNase A. Scale bar, 20 μm. (**B**) Immuno-dot blot analysis of Z-RNA in WT pBMDMs treated with RNase A upon treatment of EtOH plus IFN-β. (**C**) Representative data of quantitative real-time PCR (qPCR) analysis of *Adar1* in WT pBMDM treated with IFN-β, EtOH, or a combination of both. (**D**) Representative data of qPCR analysis of interferon-stimulated genes (*Slfn5*, *Ifih1*, *Ddx58*, and *Isg15)* in WT pBMDMs treated with JNK-IN-8 (JNKi) in the presence of EtOH plus IFN-β. *Gapdh* was used as an internal control. (**E**) RNA immunoprecipitation-qPCR analysis of *L1MdT* and *L1MdA* transcripts in Z22 immunoprecipitates relative to IgG controls in pBMDMs treated with EtOH plus IFN-β in the presence of JNKi. Enrichment was normalized to the untreated (UT) sample. Data are representative of at least three independent experiments (A to D) and represent the mean from technical replicates (E). *$p < 0.05$; **$p < 0.01$; ***$p < 0.001$; ****$p < 0.0001$. Analysis was performed using one-way ANOVA and data are shown as mean ± SEM (C to E).

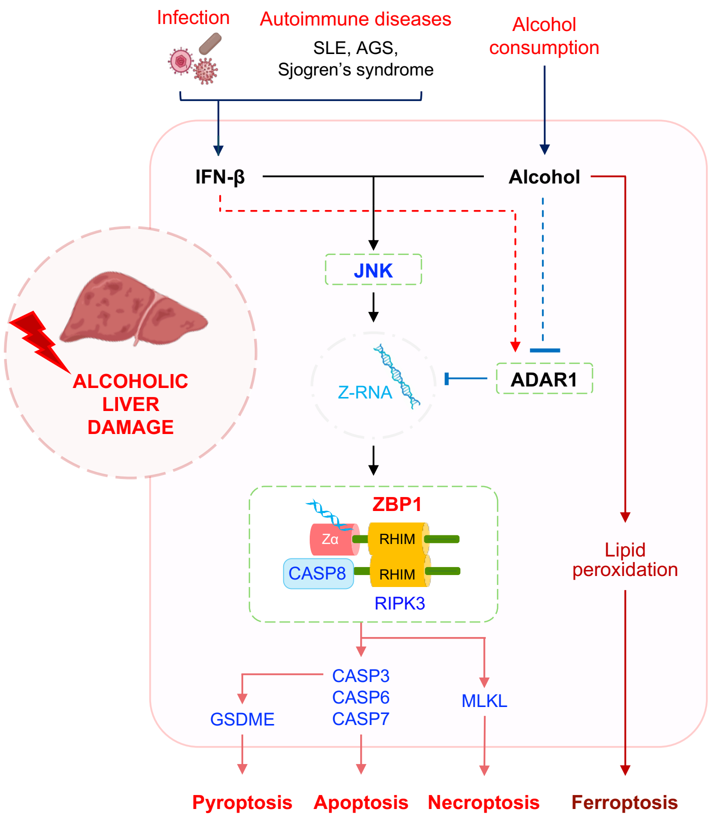

Fig. S8. A schematic diagram of alcohol and interferon exacerbating alcoholic liver disease. Alcohol and interferon activate the JNK signaling and produce Z-RNA formation, which is sensed by ZBP1, thereby driving ZBP1-dependent inflammatory cell death and liver injury.

Table S1. Gene-specific primers used in the quantitative real-time PCR

Gene	Primer sequence
Xrn1	Forward: GTTAGAAATTACTGATAGCTGG
	Reverse: GACAGGGTTTCTCTGTGTAG
Knl1	Forward: AGATCAGCCCAGGCTATACAG
	Reverse: AATAGCAAGCAGATAACATGC
Slfn5	Forward: CTCATTTGTCATTTGCTTTAGG
	Reverse: AAGTTTGCTCTGTTTGGCTC
Isg15	Forward: AGTGATGCTAGTGGTACAGAACT
	Reverse: CAGTCTGCGTCAGAAAGACCT
Eif2ak2	Forward: AGCTCCAAATAACCAAGATAC
	Reverse: CTCTGCTCTACACTCTATCTCC
L1MdA	Forward: ACATAGGGAAGCAGGCTACCC
	Reverse: GGCAAGACTCTGCTGGCAAGG
L1MdT	Forward: AAGCACAGAGGCGCTGAGGCAG
	Reverse: GACTAATTTCCTAAGTTCGGC
Ddx58	Forward: GAATGCACTCTGTAGTCCAG
	Reverse: ATAAATGAAAGTCAGCTCTCAG
Ifih1	Forward: GGAATGCCCATGAGGTATTG
	Reverse: AGCTTGCCACATTGCATTG
Gapdh	Forward: CGTCCCGTAGACAAAATGGT
	Reverse: TTGATGGCAACAATCTCCAC
Ifnb	Forward: AGGGCGGACTTCAAGATC
	Reverse: CTCATTCCACCCAGTGCT
18S rRNA	Forward: AACGGCTACCACATCCAAGG
	Reverse: GGGAGTGGGTAATTTGCGC
Adar1	Forward: GCCAAAGACAGTGGTCAACCAG
	Reverse: GAACAAGGATGTTGCTGAGGAGC

Table S2. Data on the body weight change (%) of mice in alcoholic liver disease model

Sample #	Control diet + PBS (n=9)					
	Day 0	Day 2	Day 4	Day 6	Day 8	Day 10
# 1	100	104.2453	100.4717	100	100.4717	103.3019
# 2	100	110.3448	107.3892	104.4335	108.3744	108.867
# 3	100	97.51244	104.4776	103.4826	100.995	106.9652
# 4	100	109.7938	102.0619	99.48454	103.0928	103.0928
# 5	100	100	99.49495	98.48485	98.9899	105.0505
# 6	100	100.3115	91.90031	97.50779	101.5576	102.4922
# 7	100	107.4468	98.93617	105.3191	107.0922	106.7376
# 8	100	102.9801	94.37086	101.3245	104.3046	104.6358
# 9	100	102.9221	93.83117	100	102.2727	103.2468

Sample #	Control diet + IFN-γ (n=9)					
	Day 0	Day 2	Day 4	Day 6	Day 8	Day 10
# 1	100	108.2902	109.3264	108.8083	107.772	110.3627
# 2	100	104.717	100.9434	97.64151	99.0566	94.81132
# 3	100	103.1915	99.46809	100.5319	103.7234	102.1277
# 4	100	101.5625	100	99.47917	103.125	104.1667
# 5	100	100.8811	102.6432	99.55947	101.3216	103.9648
# 6	100	102.5478	103.8217	104.4586	103.5032	104.4586
# 7	100	103.169	105.2817	104.9296	106.338	106.6901
# 8	100	102.8369	96.80851	102.8369	104.9645	106.7376
# 9	100	100.7143	97.14286	100.7143	102.1429	103.9286

Sample #	EtOH diet + PBS (n=9)					
	Day 0	Day 2	Day 4	Day 6	Day 8	Day 10
# 1	100	96.57143	92.57143	91.42857	89.71429	93.71429
# 2	100	93.83886	93.36493	87.67773	87.67773	89.09953
# 3	100	97.19101	96.06742	90.44944	88.76404	88.76404
# 4	100	98.32402	92.17877	88.82682	93.85475	93.29609
# 5	100	96.5035	94.75524	88.11189	84.61538	87.06294
# 6	100	94.89051	91.24088	86.86131	83.57664	85.76642
# 7	100	95.68345	93.88489	88.84892	84.53237	87.05036
# 8	100	98.02372	93.67589	90.90909	85.77075	89.72332
# 9	100	92.10526	90.22556	86.09023	84.58647	88.34586

Sample #	EtOH diet + IFN-γ (n=9)					
	Day 0	Day 2	Day 4	Day 6	Day 8	Day 10
# 1	100	96.74419	95.34884	83.72093	80.46512	80.93023
# 2	100	97.2093	94.88372	95.81395	94.88372	96.27907
# 3	100	95.87629	93.29897	93.29897	91.23711	96.39175
# 4	100	92.10526	90.22556	86.09023	84.58647	88.34586
# 5	100	94.77124	92.81046	85.62092	83.66013	87.2549

# 6	100	93.44262	95.90164	91.39344	88.93443	91.39344
# 7	100	96.25468	97.75281	91.7603	90.26217	91.38577
# 8	100	92.2449	93.06122	89.79592	85.30612	86.93878
# 9	100	93.10345	93.10345	88.96552	85.17241	87.93103

Table S3. Analysis of serum level in WT mice of alcoholic liver disease model

Sample #	IL-1β (pg/mL)		
	Control diet (n=5)	EtOH diet + PBS (n=8)	EtOH diet + IFN-γ (n=8)
# 1	79.85146	484.046	967.312
# 2	80.03463	884.214	1511.01
# 3	49.67438	439.19	716.946
# 4	60.04052	609.36	1167.586
# 5	55.34389	591.424	1061.48
# 6		740.212	1566.076
# 7		862.296	1384.492
# 8		692.864	1370.438

Table S4. Analysis of serum level in WT and *Ifnar1*$^{-/-}$ mice of alcoholic liver disease model

Sample #	ALT (IU/L)		IL-1β (pg/mL)	
	WT (n=14)	*Ifnar1*$^{-/-}$ (n=5)	WT (n=18)	*Ifnar1*$^{-/-}$ (n=15)
# 1	35.05757	17.39275	967.312	705.852
# 2	44.80657	9.223815	1511.01	209.79
# 3	86.26671	40.55427	716.946	534.774
# 4	67.02289	24.71258	1167.586	584.756
# 5	195.8795	3.280649	1061.48	832.87
# 6	170.7369		1566.076	549.944
# 7	114.545		1384.492	1378.648
# 8	177.334		1370.438	1021.728
# 9	69.01183		974.768	983.748
# 10	38.47583		1304.248	840.788
# 11	96.3126		1470.984	922.72
# 12	168.3205		1518.666	1037.014
# 13	41.14936		1205.744	1120.03
# 14	143.1111		1081.694	1179.91
# 15			1405.58	271.192
# 16			989.942	
# 17			1844.524	
# 18			219.078	

Table S5. Analysis of serum level in WT and *Zbp1*$^{-/-}$ mice of alcoholic liver disease model

Sample #	ALT (IU/L)		IL-1β (pg/mL)	
	WT (n=12)	*Zbp1*$^{-/-}$ (n=13)	WT (n=14)	*Zbp1*$^{-/-}$ (n=11)
# 1	170.4608	66.32452	1115.86	438.978
# 2	165.2636	62.91041	541.378	533.042
# 3	127.5838	96.5952	1012.03	318.554
# 4	101.286	22.98996	1388.178	570.44
# 5	41.37781	80.39899	1749.956	1128.362
# 6	144.6752	92.20003	974.768	1399.044
# 7	237.187	14.73297	1304.248	1040.468
# 8	96.66545	117.9117	1470.984	844.474
# 9	46.20628	7.881768	1518.666	936.26
# 10	319.4141	68.23326	1205.744	645.44
# 11	34.6663	19.92875	1081.694	171.76
# 12	263.239	68.42744	1405.58	
# 13		107.171	989.942	
# 14			1844.524	

Table S6. Analysis of serum level in WT and *Mlkl*$^{-/-}$ mice of alcoholic liver disease model

Sample #	ALT (IU/L)	
	WT (n=3)	*Mlkl*$^{-/-}$ (n=3)
# 1	115.4	68.6
# 2	134.3	154
# 3	80.2	119.3

Other Supplementary Material for this manuscript includes the following:

Data S1: RNA sequencing data presented in Figure 1C.
